# Evi1 governs Kdm6b-mediated histone demethylation to regulate the Laptm4b-driven mTOR pathway in hematopoietic progenitor cells

**DOI:** 10.1172/JCI173403

**Published:** 2024-12-16

**Authors:** Qiong Wu, Chunjie Yu, Fang Yu, Yiran Guo, Yue Sheng, Liping Li, Yafang Li, Yutao Zhang, Chao Hu, Jue Wang, Tong-chuan He, Yong Huang, Hongyu Ni, Zhiguang Huo, Wenshu Wu, Gang Greg Wang, Jianxin Lyu, Zhijian Qian

**Affiliations:** 1 Zhejiang Provincial Key Laboratory of Medical Genetics, Key Laboratory of Laboratory Medicine, Ministry of Education, School of Laboratory Medicine and Life Sciences, Wenzhou Medical University, Wenzhou, Zhejiang, China.; 2 Department of Medicine and Department of Biochemistry and Molecular Biology, UF Health Cancer Center, University of Florida, Gainesville, Florida, USA.; 3 Lineberger Comprehensive Cancer Center, University of North Carolina at Chapel Hill School of Medicine, Chapel Hill, North Carolina, USA.; 4 Department of Pathology at Geisinger Medical Center, Danville, Pennsylvania, USA.; 5 Department of Biostatistics, University of Florida, Gainesville, Florida, USA.; 6 Department of Medicine, University of Illinois at Chicago, Chicago, Illinois, USA.; 7 Department of Orthopaedic Surgery and Rehabilitation Medicine, University of Chicago, Chicago, Illinois, USA.; 8 Department of Medicine, University of Virginia, Charlottesville, Virginia, USA.; 9 Department of Pathology, Cedars-Sinai Medical Center, Los Angeles, California, USA.; 10 Department of Laboratory Medicine, Zhejiang Provincial People’s Hospital, Affiliate People’s Hospital of Hangzhou Medical College, and; 11 Laboratory Medicine of Hangzhou Medical College, Hangzhou, Zhejiang, China.

**Keywords:** Hematology, Hematopoietic stem cells, Leukemias

## Abstract

Ecotropic viral integration site 1 (EVI1/MECOM) is frequently upregulated in myeloid malignancies. Here, we present an Evi1-transgenic mouse model with inducible expression in hematopoietic stem/progenitor cells (HSPCs). Upon induction of *Evi1* expression, mice displayed anemia, thrombocytopenia, lymphopenia, and erythroid and megakaryocyte dysplasia with a significant expansion of committed myeloid progenitor cells, resembling human myelodysplastic syndrome/myeloproliferative neoplasm–like (MDS/MPN–like) disease. Evi1 overexpression prompted HSPCs to exit quiescence and accelerated their proliferation, leading to expansion of committed myeloid progenitors while inhibiting lymphopoiesis. Analysis of global gene expression and Evi1 binding site profiling in HSPCs revealed that Evi1 directly upregulated lysine demethylase 6b (Kdm6b). Subsequently, Kdm6b-mediated H3K27me3 demethylation resulted in activation of various genes, including *Laptm4b*. Interestingly, *KDM6B* and *LAPTM4B* are positively correlated with *EVI1* expression in patients with MDS. The EVI1/KDM6B/H3K27me3/LAPTM4B signaling pathway was also identified in EVI1^hi^ human leukemia cell lines. We found that hyperactivation of the LAPTM4B-driven mTOR pathway was crucial for the growth of EVI1^hi^ leukemia cells. Knockdown of Laptm4b partially rescued Evi1-induced abnormal hematopoiesis in vivo. Thus, our study establishes a mouse model to investigate EVI1^hi^ myeloid malignancies, demonstrating the significance of the EVI1-mediated KDM6B/H3K27me3/LAPTM4B signaling axis in their maintenance.

## Abstract 2

Evi1 overexpression induces MDS/MPN-like disease by activating Kdm6b/H3K27me3/Laptm4b signaling pathway

## Introduction

Myelodysplastic syndromes (MDSs) are a group of clonal hematopoietic disorders characterized by abnormal development and maturation of blood cells in bone marrow. These disorders often lead to bone marrow failure, resulting in insufficient production of mature and functional blood cells. The hallmark features of MDS include morphologic dysplasia in one or more blood cell lineages and cytopenias of the peripheral blood (1, 2). This clinical phenotype of MDS is nonspecific and can overlap with various other benign or malignant conditions, such as myeloproliferative neoplasm (MPN). These disorders often exhibit hematopoietic dysplasia with increased proliferation of monocytes, neutrophils, or platelets (1, 3, 4). Approximately 30% of patients diagnosed with MDS ultimately develop acute myeloid leukemia (AML) (5).

*Evi1* was first identified as a common site of ecotropic viral integration in mice with retrovirally induced myeloid malignancies (6). The human *EVI1* (*MECOM*) gene is located on chromosome 3q26, and multiple isoforms of *EVI1* are encoded in the MECOM locus (7). Rearrangements of chromosome 3q26, which lead to upregulation of *EVI1*, frequently occur in myeloid malignant diseases including MDS, AML, and chronic myeloid leukemia (CML) (8–10). MDS, AML, and CML with inv(3)/t(3;3) rearrangements often present similar pathological features with poor prognosis (8, 11, 12). It was reported that chromosome rearrangements cause overexpression of *EVI1* due to relocation of enhancers, including GATA binding protein 2 (GATA2) enhancer in inv(3)/t(3;3) (q21q26) (13, 14) and MYC super-enhancer in t(3;8) (q26;q24) close to the *EVI1* gene (15). *EVI1* overexpression can occur in MDS patients without chromosome 3 rearrangements. *EVI1* upregulation is detected in approximately 8%–10% of MDS/AML and 30% of advanced CML; however, the mechanism that results in *EVI1* overexpression remains unclear (16). Additionally, high expression of *EVI1* is also detected in a subgroup of MDS/MPN (17). MDS/AML with *EVI1* overexpression often exhibits dysplasia of erythrocyte and megakaryocytic lineages (8, 12, 18).

Evi1 is essential for the maintenance of long-term hematopoietic stem cells (LT-HSCs) in mice (19). Introduction of *EVI1* via retroviral expression in hematopoietic stem/progenitor cells (HSPCs) led to the development of MDS in mice, while these mice were unable to develop AML with long latencies (20), suggesting that *EVI1* alone is insufficient to induce leukemia in mice. However, other studies also showed that *EVI1* overexpression can induce AML in mice (13, 21–23), possibly due to additional mutations resulting from retroviral insertion or prolonged disease progression in mice. Moreover, the degree of *EVI1* overexpression in vivo may also contribute to the varying phenotypes observed in mouse models of *EVI1* overexpression.

Studies have reported that EVI1 exerts control over cell proliferation, apoptosis, cell differentiation, and the cell cycle of hematopoietic progenitor cells through various mechanisms (24, 25). However, the molecular mechanisms underlying the role of EVI1 overexpression in leukemogenesis in vivo remain incompletely understood, and targeted therapies for MDS/AML patients with EVI1 overexpression are currently lacking.

In this study, we present a novel transgenic mouse model allowing conditional activation of Evi1 expression at low levels in HSPCs. These Evi1-transgenic mice developed a disease resembling myelodysplastic syndrome/myeloproliferative neoplasm (MDS/MPN), characterized by anemia and thrombocytopenia, accompanied by an expansion of myelopoiesis and suppressed lymphopoiesis. Erythroid and megakaryocyte dysplasia was also observed. Further characterization of HSPCs revealed that *Evi1* overexpression led to a reduction in HSCs and an expansion of myeloid progenitor cells. Through integrative analysis of gene expression profiling and EVI1 binding sites, we identified lysine demethylase 6b (Kdm6b) as a direct target of Evi1 in primary HSPCs in mice. Further investigations demonstrated that Evi1 regulated Kdm6b-mediated H3K27me3, resulting in the upregulation of multiple genes, including *Laptm4b*. Both *KDM6B* and *LAPTM4B* expression exhibited positive correlations with *EVI1* expression in patients with MDS. We observed upregulation of both KDM6B and LAPTM4B in EVI1^hi^ leukemia cells compared with EVI1^lo^ leukemia cells. In addition, LAPTM4B stimulated the mTOR pathway in EVI1^hi^ leukemia cells. Moreover, KDM6B inhibitor and LAPTM4B knockdown significantly inhibited growth and induced apoptosis of EVI1^hi^ leukemia cell lines. Furthermore, Laptm4b knockdown partially reversed Evi1-induced aberrant hematopoiesis in vivo. Our findings highlight the presence of the EVI1/KDM6B/H3K27me3/LAPTM4B signaling axis in *EVI1*-overexpressed myeloid malignancies and suggest the therapeutic potential of inhibition of the KDM6B/H3K27me3/LAPTM4B signaling axis in patients with EVI1^hi^ malignancies.

## Results

### Evi1 upregulation induces MDS/MPN–like disease in the mice.

**Figure 1:**
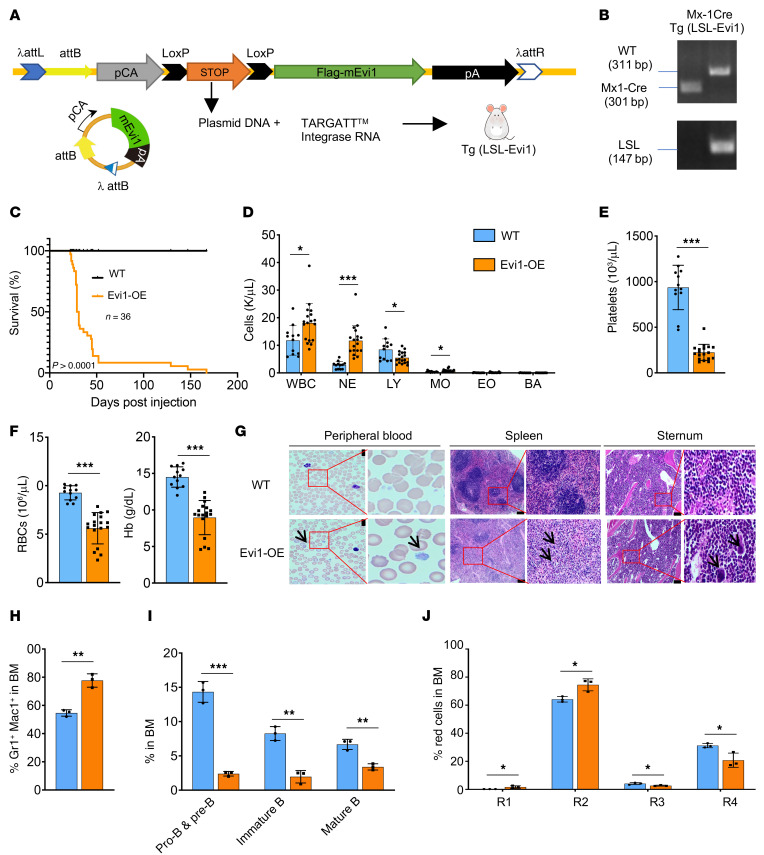
Evi1 upregulation induces MDS/MPN–like disease in mice. (**A**) Schematic illustration of conditional Evi1-induced mouse model. (**B**) PCR analysis of the induction of Evi1 (LSL) and WT alleles among genomic DNA in BM cells from Mx1-Cre Tg (LSL-Evi1) mice after poly(I:C) injection. (**C**) Kaplan-Meier survival analysis of Evi1-OE mice and WT mice after multiple injections of poly(I:C) (50 mg per kg body weight). *n* = 36 per cohort, log-rank test. (**D**–**F**) Absolute numbers of white blood cells (WBC), neutrophils (NE), lymphocytes (LY), monocytes (MO), eosinophils (EO), and basophils (BA) (**D**) and platelets (**E**), as well as red blood cells (RBCs) and concentration of hemoglobin (Hb) (**F**), in peripheral blood (PB) from Evi1-OE mice (*n* = 12) and WT mice (*n* = 18). (**G**) Representative histologic analysis of PB smear (left) as well as hematoxylin and eosin–stained spleen (middle) and sternum (right) from the mice indicated. Scale bars: 10 µm (peripheral blood); 100 µm (spleen); 50 µm (sternum). Relative magnification of these images is ×4. (**H**–**J**) Analysis of frequency of Gr1^+^Mac1^+^ myeloid cells (**H**), B cells (**I**), and red cells (**J**) in BM cells from WT and Evi1-OE mice 3 weeks after poly(I:C) injection. *n* = 3 per group. Data are representative of at least 2 independent experiments and are presented as mean ± SD; 2-tailed Student’s *t* test, or log-rank (Mantel-Cox) test for survival curve. *P < 0.05, **P < 0.01, ***P < 0.001.

To mimic the effects of EVI1 activation in patients with myeloid malignancies, we generated a conditional Evi1-transgenic model in the B6 background strain using the TARGATT system (26). In these conditional Evi1-transgenic mice, Tg (LSL-Evi1), a knockin of a FLAG-tagged mouse Evi1 transgene (Tg) with an extra exon containing a stop codon flanked by LoxP (LoxP-stop-LoxP [LSL]) recombination sequences in direct sequence orientation (Figure 1, A and B), was specifically integrated into the Rosa26 locus, supporting a stable expression of the *Evi1* gene upon inducing the expression of Cre recombinase. To induce Evi1 transgene expression specifically in HSPCs, we generated cohorts of Mx1-Cre Tg (LSL-Evi1) by crossing Mx1-Cre transgenic mice with Tg (LSL-Evi1) mice, in which Evi1 expression can be induced by poly(I:C). Accordingly, quantitative reverse transcriptase PCR (RT-qPCR) analysis showed a 2- to 3-fold increase of *Evi1* mRNA in Lin^–^c-Kit^+^Sca1^+^ cells (LSKs) from Mx1-Cre Tg (LSL-Evi1) after induction of Evi1 expression by poly(I:C) (referred to as EVI1-OE [overexpressed] hereafter) (Supplemental Figure 1A; supplemental material available online with this article; https://doi.org/10.1172/JCI173403DS1). Approximately 90% of Evi1-OE mice became moribund within 50 days (Figure 1C). Analysis of hematologic parameters in the moribund mice revealed that the mice overexpressing Evi1 exhibited a significant decrease in red blood cells (RBCs), hemoglobin levels (Figure 1F), and platelet counts (Figure 1E) while showing a slight increase in white blood cells (Figure 1D) as compared with age-matched control mice. The majority of Evi1-OE mice exhibited multilineage dysplasia characteristic of MDS/MPN (27). Specially, erythroid dysplasia, such as red cell polychromasia, and megakaryocyte dysplasia, including the presence of large hypogranular platelets, were observed in peripheral blood (PB) from Evi1-OE mice (Figure 1G, left). Spleen specimens from Evi1-OE mice (Figure 1G, middle) exhibited reduced lymphoid tissue and an increase in non-lymphoid hematopoietic cells. Both erythroid and myeloid precursors showed an increase in abundance. Dysplastic megakaryocytes, characterized by hypolobated nuclei, were frequently observed in Evi1-OE mice. In bone marrow (BM) specimens from moribund mice (Figure 1G, right), an increase in megakaryocytes was observed. Some of these megakaryocytes displayed hypolobated nuclei and emperipolesis. Erythroid precursors showed a relatively decreased abundance, while myeloid precursors were relatively increased. Likewise, flow cytometric analysis revealed that Gr1^+^Mac1^+^ mature myeloid cells were significantly increased while B cell processor cells including pro–B/pre–B cells and immature and mature B cells were all decreased in the BM (Figure 1, H and I) and spleen (Supplemental Figure 1, B and C) in Evi1-OE mice compared with WT mice. Evi1-transgenic mice had a significantly increased proportion of proerythroblasts (R1, Ter119^lo^CD71^hi^) and basophilic erythroblasts (R2, Ter119^hi^CD71^hi^) and a decreased proportion of late erythroblasts (R3, Ter119^hi^CD71^med^, and R4, Ter119^hi^CD71^lo^) in BM and spleen (Figure 1J and Supplemental Figure 1D). These results indicate that *Evi1* overexpression led to an enhanced myelopoiesis, while it blocks lymphopoiesis and erythropoiesis. Collectively, our findings demonstrate that increased expression of *Evi1* in vivo leads to the development of MDS/MPN–like disease, recapitulating the characteristic features observed in MDS/MPN patients with high *EVI1* expression.

### Evi1 upregulation results in a decrease of HSCs but an expansion of myeloid lineage–committed progenitor cells.

**Figure 2:**
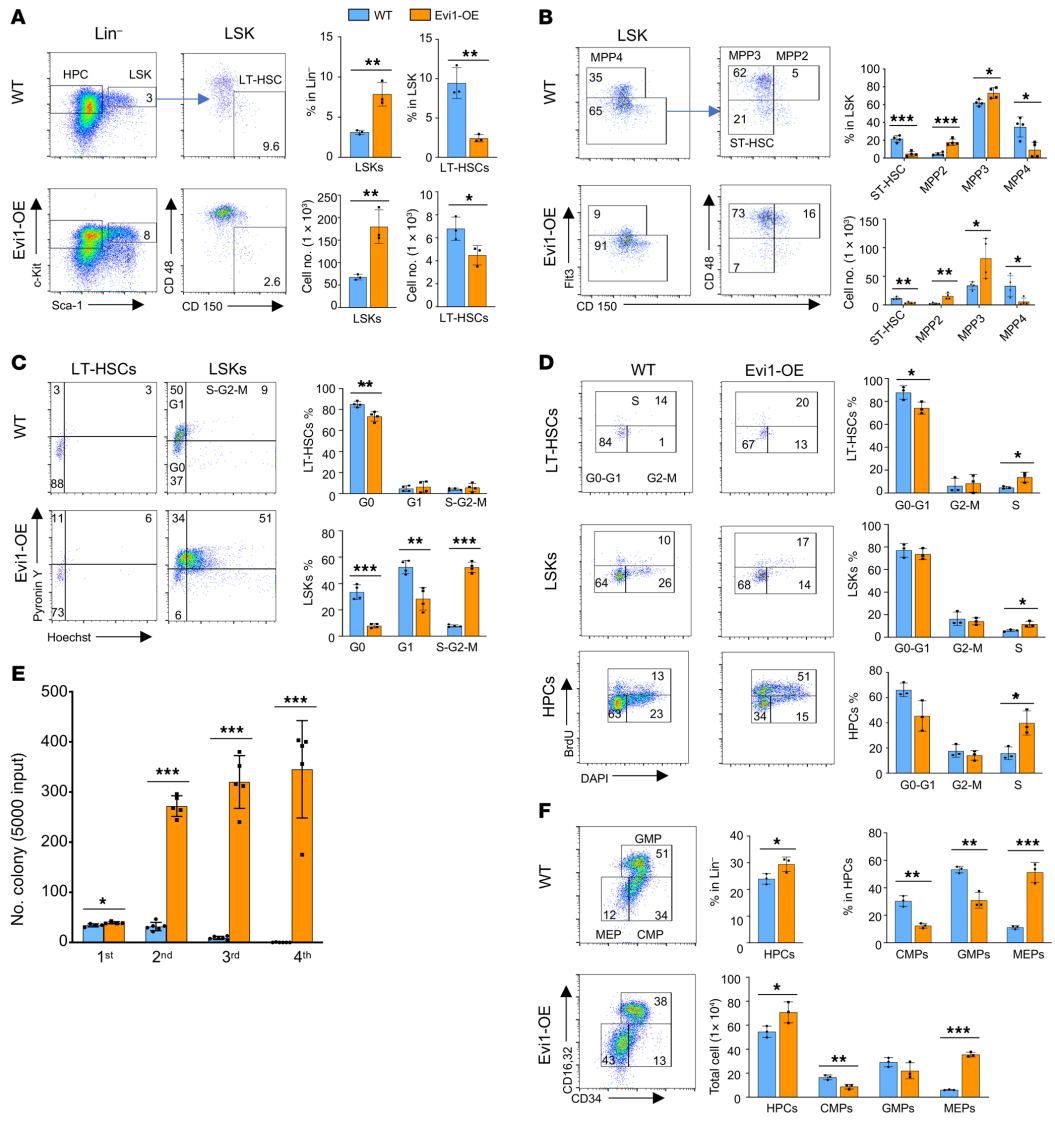
Activation of Evi1 leads to a decreased HSC quiescence. (**A**) Flow cytometric analysis of the frequency and the total number of LSKs and LT-HSCs in WT and Evi1-OE mice. *n* = 3 per group. (**B**) Frequency and total number of ST-HSC, MPP2, MPP3, and MPP4 in BM from WT and Evi1-OE mice. *n* = 4 per group. (**C**) Left panel: Flow cytometric analyses of the G_0_–G_1_ cell cycle status in LSKs, labeled with pyronin Y and Hoechst. Right panel: The histogram depicts the cell cycle status of LSKs and LT-HSCs in WT and Evi1-OE mice. *n* = 4 per group. (**D**) Representative flow cytometry plots (left panel) and quantification of the frequency of cells in different cell cycle (right panel) in LT-HSCs (top), LSKs (middle), and HPCs (bottom). *n* = 3 per group. (**E**) Quantification of colony number for serial colony-forming assay; 5,000 cells input for each round. (**F**) Representative flow cytometry plots (left panel) and quantification of the frequency and total cell number for HPCs and subsets of myeloid progenitors including CMPs, GMPs, and MEPs in WT and Evi1-OE mice. *n* = 3 per group. All data are representative of at least 2 independent experiments and are expressed as mean ± SD; 2-tailed Student’s *t* test. *P < 0.05, **P < 0.01, ***P < 0.001.

During normal hematopoiesis, LT-HSCs possess the ability to self-renew and differentiate into multipotent progenitor (MPP) cells, which further specialize into distinct blood cell lineages (28). To examine how *Evi1* upregulation leads to an aberrant hematopoiesis, we examined the HSPC compartments by flow cytometry 3 weeks after activation of *Evi1* expression by poly(I:C) injection. Notably, the total cell number and frequency of LT-HSCs (Lin^–^c-Kit^+^Sca1^+^CD48^–^CD150^+^) decreased dramatically in Evi1-OE mice as compared with the control littermates (Figure 2A). In contrast, both the frequency and the total number of LSKs, a stem cell–enriched population, were significantly increased in Evi1-OE mice (Figure 2A). MPP2 and MPP3 are distinct myeloid-biased MPP subsets that work together with lymphoid-primed MPP4 cells to control blood production (28). We found that the total number and frequency of MPP2 and MPP3 were significantly increased in Evi1-OE mice, while short-term HSCs (ST-HSCs) and MPP4 cells were dramatically decreased in the Evi1-OE group as compared with the control mice (Figure 2B). Together, these data suggest that *Evi1* overexpression promotes the expansion of myeloid-committed progenitor cells while it inhibits lymphoid-committed progenitors. However, *Evi1* overexpression reduced both LT-HSCs and ST-HSCs.

### Evi1 overexpression promotes exit of quiescence of LT-HSCs and the proliferation of HSPCs.

We next determined whether Evi1 overexpression affects the cell cycle status of LT-HSCs and LSKs by assessing RNA and DNA content through the use of staining with pyronin Y and Hoechst 33342, as previously described (29). The G_0_ phase of LT-HSCs and LSKs decreased significantly and the G_1_ phase of LSKs decreased while the G_2_–S–M phases of LSKs increased in Evi1-OE mice, as compared with control littermates (Figure 2C). We further analyzed the cell cycle dynamics of HSCs and hematopoietic progenitor cells (HPCs) in mice. The S phase of LSKs, LT-HSCs, and HPCs increased significantly in Evi1-OE mice compared with control mice (Figure 2D). Thus, these data provide compelling evidence that Evi1 overexpression facilitates the transition of LT-HSCs from a quiescent state to an actively cycling state, as well as the proliferation of HSPCs. This observation is further supported by the results of the serial replating assay, wherein HSPCs from Evi1-OE mice gave rise to a significantly higher number of total colony-forming units compared with HSPCs from control mice during the secondary, third, and fourth replating (Figure 2E). These findings strongly indicate that HSPCs with Evi1 overexpression possess an augmented capacity for self-renewal and proliferation when compared with their control counterparts. However, Evi1 overexpression does not affect the survival of LSKs, LT-HSCs, and HPCs in mice (Supplemental Figure 1, E–G).

We further characterized myeloid progenitor cells in Evi1-OE and control mice 3 weeks after poly(I:C) injection. The frequency of common myeloid progenitors (CMPs) and granulocyte-monocyte progenitors (GMPs) was significantly decreased, while the frequency of HPCs and megakaryocyte-erythroid progenitors (MEPs) was significantly increased, in Evi1-transgenic mice compared with control mice. Additionally, the total number of CMPs, but not GMPs, was decreased, whereas the total number of HPCs and MEPs was increased, in Evi1-transgenic mice (Figure 2F). These findings suggest that Evi1 overexpression may promote GMP differentiation but block MEP differentiation.

### Evi1 overexpression–induced MDS/MPN is transplantable.

**Figure 3:**
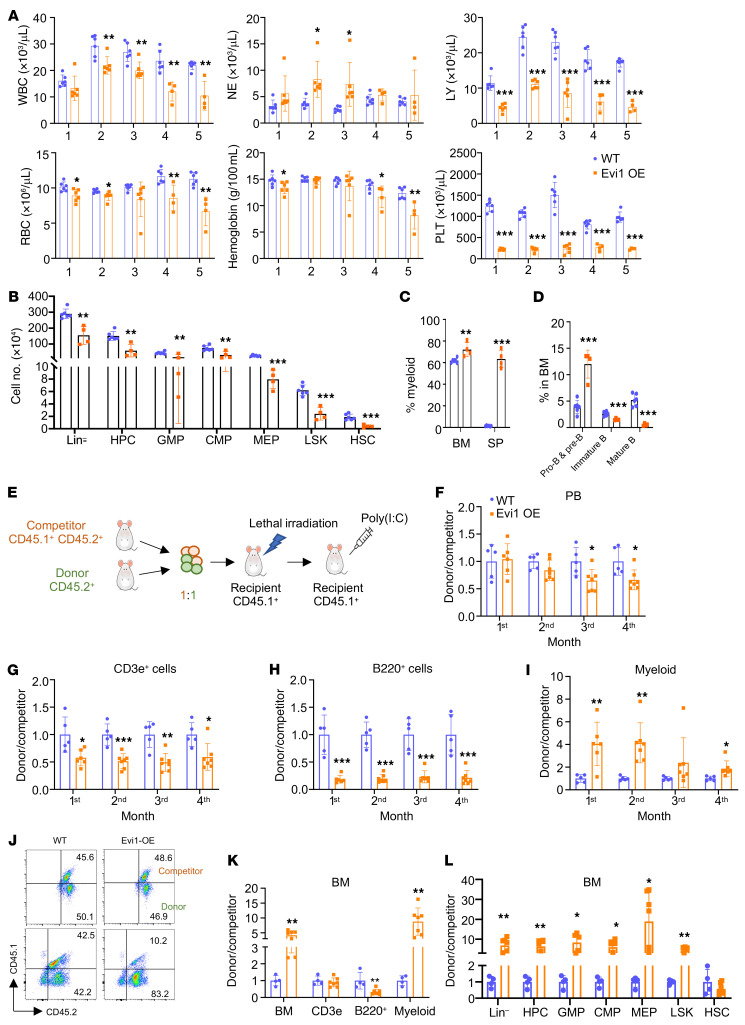
Evi1-induced MDS/MPN is transplantable. (**A**) Absolute numbers of WBCs, NEs, LYs, and RBCs as well as concentration of Hb and platelets (PLT) in PB from Evi1-OE and WT recipient mice. *n* = 6 for WT group, *n* = 4 for Evi1-OE group. (**B**) Flow cytometric analysis of the total number of Lin^–^ cells, HPCs, and subsets of myeloid progenitors including CMPs, GMPs, and MEPs in the mice indicated in **A**. (**C**) Frequency of Gr1^+^Mac1^+^cells in BM and spleen from the mice indicated in **A**. (**D**) Frequency of the subsets of B cells in BM cells. (**E**) Schematic illustration of the competitive transplantation assay. (**F**) The relative ratio of donor-derived cells (CD45.1^–^CD45.2^+^) to competitor-derived cells (CD45.1^+^CD45.2^+^) in PB from the recipient mice. (**G**–**I**) The relative ratio of donor-derived cells to competitor-derived cells in CD3e^+^ cells (**G**), B220^+^ cells (**H**), and myeloid cells (**I**) in PB. *n* = 5 for WT group, *n* = 6–7 for Evi1-OE group in **F**–**I**. (**J**) Representative flow cytometry plots for the donor and competitor cells before injection and at the fourth month of transplantation. (**K**) The relative ratio of donor-derived cells to competitor-derived cells in the total BM cells and different lineage cells in BM. (**L**) The relative ratio of donor-derived cells to competitor-derived cells in different populations as indicated in BM. *n* = 4 for WT group, *n* = 7 for Evi1-OE group in **K** and **L**. All data are representative of at least 2 independent experiments and are expressed as mean ± SD; 2-tailed Student’s *t* test. *P < 0.05, **P < 0.01, ***P < 0.001.

To investigate the transplant ability of *Evi1* overexpression–induced MDS/MPN, BM cells isolated from 2 diseased Evi1-transgenic mice and control mice were transplanted into lethally irradiated WT recipient mice. Remarkably, the recipient mice overexpressing *Evi1* displayed a significant decrease in lymphoid cells and platelets, accompanied by an increase in neutrophils (Figure 3A). Flow cytometry analysis verified these changes (Figure 3, C and D, and Supplemental Figure 2, A–C). However, 3 months after transplantation, the neutrophil count gradually decreased. After 5 months of transplantation, the total BM cell counts were comparable between the Evi1-overexpressed recipients and control recipients (Supplemental Figure 2B). Upon further examination of HSPCs in these mice, we observed a significant reduction in the total number of HSCs, LSKs, HPCs, and subsets of myeloid progenitors in the Evi1-overexpressed recipient mice compared with the control recipient mice (Figure 3B). Intriguingly, none of the Evi1-overexpressed recipients exhibited signs of morbidity 5 months after transplantation. These findings suggest that HSPCs from primary Evi1-transgenic mice can regenerate MDS/MPN disease in recipient mice, albeit with less severe phenotypes.

Due to the activation of Mx1-Cre transgene expression by poly(I:C), gene expression is induced not only in hematopoietic cells but also in nonhematopoietic cells such as the BM stromal compartment. To explore the potential contribution of the BM microenvironment to Evi1-induced MDS/MPN disease in mice, we transplanted BM cells from Evi1-OE or WT mice (CD45.2^+^) into lethally irradiated WT synergetic recipient mice (CD45.1^+^) (Supplemental Figure 3A). Engraftment efficiency was comparable between Evi1-OE and WT BM cells, with over 90% replacement of recipient BM cells by donor BM cells (Supplemental Figure 4, A and B). Poly(I:C) was administered to the chimeric mice to induce Evi1 overexpression 4 weeks after transplantation. As depicted in Supplemental Figures 3 and 4, the Evi1-overexpressed recipient mice developed a similar MDS/MPN–like disease characterized by decreased lymphocyte (LY), RBC, hemoglobin, and platelet counts in PB, along with an increased neutrophil (NE) count (Supplemental Figure 3, B–G). Additionally, there was a notable reduction in LT-HSCs and an increase in MEPs in the recipient mice with Evi1 overexpression compared with the control mice (Supplemental Figure 4, I and K). Notably, after 5 months of transplantation, none of the Evi1-overexpressed recipient mice exhibited signs of morbidity, indicating that the Evi1-overexpressed recipient mice developed the disease with a significantly longer latency compared with the primary Evi1-transgenic mice (Supplemental Figure 4, C–K).

To assess the function of the Evi1-overexpressing HSPCs under competitive stress, we performed a competitive assay, in which CD45.1^+^ recipient mice received lethal irradiation followed by transplantation of CD45.2^+^ BM cells from Tg (LSL-Evi1) or Mx1-Cre Tg (LSL-Evi1) mice, along with CD45.2^+^CD45.1^+^ BM cells as competitor (Figure 3E). After 1 month of transplantation, poly(I:C) injection was administered to induce the overexpression of Evi1. PB from the recipient mice was collected monthly and analyzed by flow cytometry to determine the contribution of donor cells (CD45.2^+^) versus competitor cells (CD45.2^+^CD45.1^+^) to hematopoiesis (Figure 3J). In line with previous BM transplantation models, we observed a gradual decrease in the proportion of blood cells that originated from Evi1-overexpressing HSPCs in PB compared with WT recipients (Figure 3F). Specifically, CD3e^+^ cells and B220^+^ cells derived from Evi1-overexpressing HSPCs showed a significant decrease in representation in the PB over a 4-month transplantation period, while myeloid cells exhibited a notable increase at the first and second months of transplantation, followed by a gradual decline (Figure 3, G–I). To assess the donor-derived HSPCs, we analyzed BM cells from recipient mice using flow cytometry at the fourth month after transplantation. Interestingly, compared with the WT group, the recipient mice in the Evi1-OE group exhibited a higher proportion of total BM cells and myeloid cells, along with a decrease in B220^+^ cells derived from donor HSPCs (Figure 3K). Moreover, Evi1-overexpressing HSPCs outcompeted the competitor HSPCs and generated a higher number of HPCs with a relatively lower number of HSCs (Figure 3L). Together, these findings suggest that Evi1-overexpressing myeloid progenitor cells gained a growth/repopulation advantage over the WT HSPCs, promoting clonal dominance of Evi1^hi^ myeloid progenitor cells in vivo in mice, recapitulating the process of MDS/MPN development observed in patients.

### Evi1 binds to the promoter of Kdm6b and regulates its expression.

**Figure 4:**
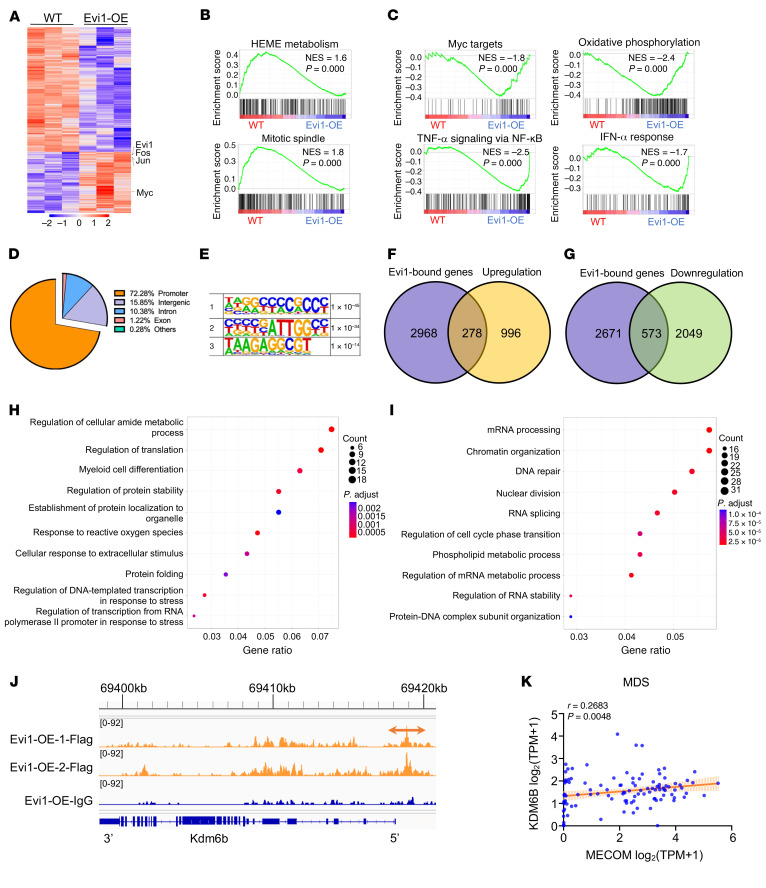
Transcriptional analysis of Evi1 overexpression–induced differentially expressed genes in hematopoietic stem and progenitor cells. (**A**) Heatmap of differentially regulated genes in LSKs isolated from WT and Evi1-OE mice. *n* = 3 for each group. (**B**) GSEA plots showing, respectively, a negative association with “heme metabolism” and “mitotic spindle” in Evi1-OE LSKs compared with WT LSKs. (**C**) GSEA plots showing a positive association with “Myc target V2,” “TNF-α signaling via NF-kB,” “oxidative phosphorylation,” and “IFN-α response” pathways in Evi1-OE LSKs compared with WT LSKs. (**D**) The genomic distribution of Evi1 binding sites in LSKs identified by CUT&RUN-seq analysis. (**E**) The top 3 Evi1-binding consensus sequences identified by CUT&RUN-seq analysis in Lin^–^c-Kit^+^ cells are shown. (**F** and **G**) Venn diagram showing the overlap between Evi1-enriched genes in CUT&RUN-seq assay and the genes significantly upregulated (**F**) or downregulated (**G**) in Evi1-OE LSKs based on RNA-Seq result (*P* < 0.05). (**H**) Gene Ontology analysis of overlapped genes in **F**. (**I**) Gene Ontology analysis of overlapped genes in **G**. (**J**) IGV peak visualization of Evi1 binding sites within the transcript of *Kdm6b* in WT or Evi1-overexpressing Lin^–^c-Kit^+^ mouse BM cells identified by CUT&RUN-seq analysis. (**K**) Correlation between *EVI1* (*MECOM* in the database) and *KDM6B* in patients with MDS (GEO GSE114922). *P* value was calculated by Spearman’s *r* correlation.

To investigate the mechanism underlying the Evi1-OE–mediated development of MDS/MPN–like disease, we performed global gene expression profiling of a stem cell–enriched population (LSKs) isolated from the recipients that received BM cells from Mx1-Cre Tg (LSL-Evi1) or Tg (LSL-Evi1) mice 4 weeks after poly(I:C) injection. In Evi1-overexpressing LSKs compared with the control LSKs, 1,274 genes were differentially upregulated, while 2,622 genes were downregulated (Figure 4A). c-Fos and c-Jun form an activator protein-1 (AP-1) complex and can interact with EVI1 (30). We found that both those transcription factors were significantly upregulated in Evi1-OE LSKs (Figure 4A). Additionally, MYC, which is positively correlated with EVI1 (24, 31, 32), is also upregulated in Evi1-OE LSKs (Figure 4A). The gene set enrichment analysis (GSEA) revealed that the set of genes downregulated in Evi1-overexpressing LSKs showed enrichment for a gene set encoding products associated with heme metabolism and mitotic spindle signaling pathways (Figure 4B).

Notably, the set of genes upregulated in Evi1-overexpressing LSKs showed enrichment for a gene set encoding products associated with Myc targets and multiple pathways, including the TNF-α signaling pathway, oxidative phosphorylation, and the IFN-α response signaling pathway (Figure 4C). Upregulation of c-Myc and oxidative phosphorylation may contribute to Evi1 overexpression–activated proliferation of HSPCs. Tight regulation of heme metabolism is required for normal erythropoiesis (33). Evi1 overexpression–induced downregulation of heme metabolism may partially account for the development of anemia in Evi1-OE mice. Elevated levels of TNF-α have been observed in the BM of patients with MDS (34–36), and this can contribute to the dysregulation of hematopoiesis and disease progression (37, 38). TNF-α has been shown to inhibit the differentiation and maturation of erythroid cells (39). Thus, upregulation of the TNF-α signaling pathway may also partially mediate the role of Evi1 overexpression in erythropoiesis and impaired hematopoiesis. Thus, these Evi1 overexpression–mediated transcriptional changes indicated that the activation of Evi1 perturbed the function of HSPCs through multiple molecular mechanisms.

The global Evi1 binding targets in primary HSPCs in vivo have not been reported yet. We next performed a cleavage under targets and release using nuclease followed by sequencing (CUT&RUN-seq) experiment with isolated WT and Evi1-overexpressing Lin^–^c-Kit^+^ cells to identify the potential direct target genes of Evi1 in HSPCs in vivo. We identified 6,139 CUT&RUN-seq peaks in Lin^–^c-Kit^+^ cells. Analysis of peak locations relative to gene annotations revealed a broad distribution of Evi1 binding sites throughout the transcripts with a bias toward regions proximal to the transcription start site. The majority of the Evi1 binding peaks (72%) were located in the promoter regions (Figure 4D). A total of 3,246 Evi1-bound genes were identified in Lin^–^c-Kit^+^ cells. Three Evi1 consensus binding motifs, NNRGCCCCGCCC, YBYYGATTGGCY, and WAAGAGGCGT, were identified in the regulatory region of target genes influenced by Evi1 in Lin^–^c-Kit^+^ BM cells, and the top 3 enriched Evi1 consensus binding sequences are shown in Figure 4E. Notably, a very similar sequence, srrrdrvykaGAAAGrkGmAt, was also observed in a human EVI1-binding consensus motif predicted by the JASPAR database, suggesting that the Evi1 binding sites in a set of genes may be conserved among species.

Of the 3,896 total differentially expressed genes (DEGs) in Evi1-overexpressing LSKs, 278 upregulated genes and 573 downregulated genes contained predicted Evi1 binding sites proximal to their promoter regions (Figure 4, F and G). We performed Gene Ontology (GO) analysis for the DEGs. The upregulated DEGs with Evi1 binding sites showed an enrichment in gene sets involving mitotic regulation of translation, response to oxidative stress, myeloid cell differentiation, and protein folding (Figure 4H), while the downregulated DEGs with Evi1 binding sites showed an enrichment in gene sets involving chromosome segregation, chromatin organization, mRNA processing, DNA repair, the RNA splicing pathway, and regulation of RNA stability (Figure 4I). Kdm6b, a member of JmjC domain–containing histone demethylases that specifically removes methyl groups from H3K27me3 to enable the activation of its target genes (40), stands out, as it has an important role in human diseases (41–43). We observed a robust Evi1 binding proximal to the *Kdm6b* promoter (Figure 4J). Notably, *KDM6B* was positively correlated with *EVI1* expression in patients with MDS (Gene Expression Omnibus [GEO] database, GSE114922) **(**Figure 4K). Collectively, these data suggest that *Evi1* directly regulates the expression of a set of genes involving multiple molecular pathways in hematopoietic progenitor cells. Additionally, Kdm6b-mediated pathways may partially contribute to Evi1 overexpression–induced myeloid progenitor expansion.

### Evi1 overexpression promotes Kdm6b-mediated H3K27me3 demethylation.

**Figure 5:**
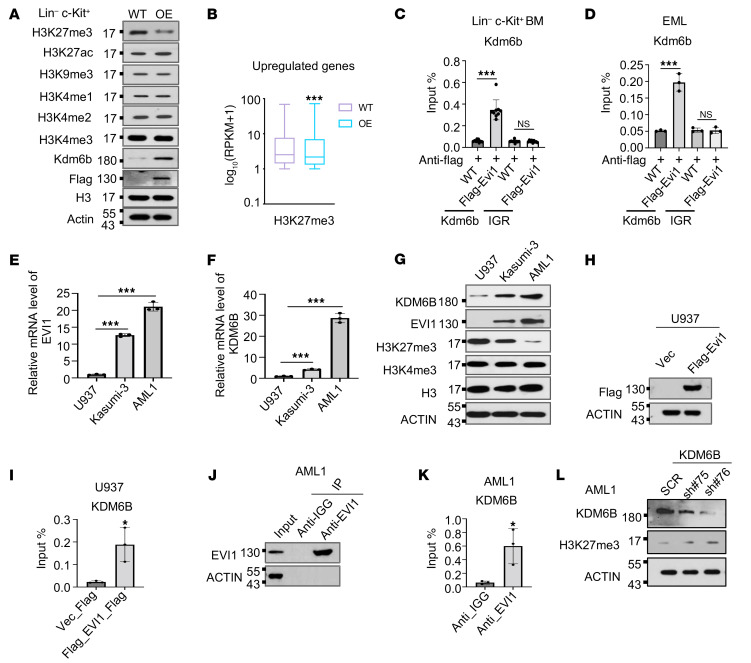
Evi1 regulates H3K27me3 level through Kdm6b. (**A**) Western blot (WB) analysis showing H3 modifications and Kdm6b expression using Lin^–^c-Kit^+^ cells from WT and Evi1-overexpressing mice. Actin and H3 were used as loading controls. (**B**) Box plot showing metagene analysis for the H3K27me3 level at the promoter regions of upregulated genes in Evi1-overexpressing Lin^–^c-Kit^+^ cells. (**C** and **D**) ChIP-qPCR analyses indicate that Evi1 directly binds to the promoter region of *Kdm6b* in Lin^–^c-Kit^+^ mouse BM cells (**C**) and erythroid myeloid lymphoid (EML) cells (**D**). (**E** and **F**) Assessment of the expression level of *EVI1* and *KDM6B* at mRNA level in U937, Kasumi-3, and AML1 cells by RT-qPCR. (**G**) Western blot assessed the protein levels of EVI1, KDM6B, H3K27me3, and H3K4me3 in U937, Kasumi-3, and AML1 cells. (**H**) WB analysis confirming the protein expression of FLAG-tagged EVI1 in U937 cells. (**I**) ChIP-qPCR analysis indicates that exogenous EVI1 directly binds to the promoter region of *KDM6B* in U937 cells. (**J**) WB analysis showing the EVI1 enrichment for the ChIP-qPCR analysis in AML1 cells. (**K**) ChIP-qPCR analysis indicates that endogenous EVI1 directly binds to the promoter region of *KDM6B* in AML1 cells. (**L**) WB analysis shows the downregulation of KDM6B and upregulation of H3K27me3 in AML1 cells transduced with *KDM6B* shRNAs. Data are representative of at least 2 independent experiments. In **B**, median values are indicated by the line within the box plot (minimum to maximum whiskers). *P* value was calculated by a 2-tailed Mann-Whitney test. In **C**–**F**, **I**, and **K**, data are presented as mean ± SD, with ordinary 1-way ANOVA with Dunnett’s multiple-comparison test used for **C**–**F** and 2-tailed Student’s *t* test for **I** and **K**. *P < 0.05, ***P < 0.001.

Upon discovering that Kdm6b is a direct downstream target of Evi1, we became intrigued by the potential relationship between Evi1 and abnormal histone modifications in HSPCs and how it might influence the functions of HSPCs. We evaluated the protein levels of active histone marks, such as H3K27ac and H3K4me1/2/3, as well as inactive marks including H3K27me3 and H3K9me3, in Lin^–^c-Kit^+^ mouse BM cells with or without Evi1 overexpression. Notably, Evi1 overexpression resulted in a substantial increase in Kdm6b protein levels while simultaneously reducing the abundance of H3K27me3 in Lin^–^c-Kit^+^ mouse BM cells (Figure 5A). Supporting our observations, metagene analysis of the H3K27me3 CUT&RUN-seq results provided further evidence of a global decrease in H3K27me3 enrichment specifically at the promoter regions of target genes following Evi1 overexpression (Figure 5B). By chromatin immunoprecipitation followed by RT-qPCR (ChIP-qPCR), we further verified the direct binding of Evi1 to the promoter region of Kdm6b, aligning with the findings from our Evi1 CUT&RUN-seq analysis (Figure 5, C and D). Given that EVI1 is upregulated in a subset of patients with AML associated with poor survival, we sought to determine whether this regulatory mechanism also occurs in AML cells. Strikingly, both the transcription and protein levels of KDM6B were significantly elevated in the EVI1^hi^ Kasumi-3 and AML1 cells compared with the EVI1^lo^ U937 cells (Figure 5, E–G). In contrast, the protein abundance of H3K27me3 exhibited a significant reduction in Kasumi-3 and AML1 cells compared with U937 cells (Figure 5G). Moreover, we verified the direct binding of both exogenous and endogenous EVI1 to the promoter region of KDM6B in U937 and AML1 cells (Figure 5, H–K). Furthermore, by using 2 specific shRNAs to knock down KDM6B expression, we observed a substantial increase in the protein levels of H3K27me3 (Figure 5L). Collectively, these compelling findings demonstrate that Evi1 overexpression selectively decreases the global level of H3K27me3 in both mouse progenitor cells and human AML1 cells in a KDM6B-dependent manner.

### Transcriptome-wide analysis identifies Laptm4b as a functional mediator of Evi1.

**Figure 6:**
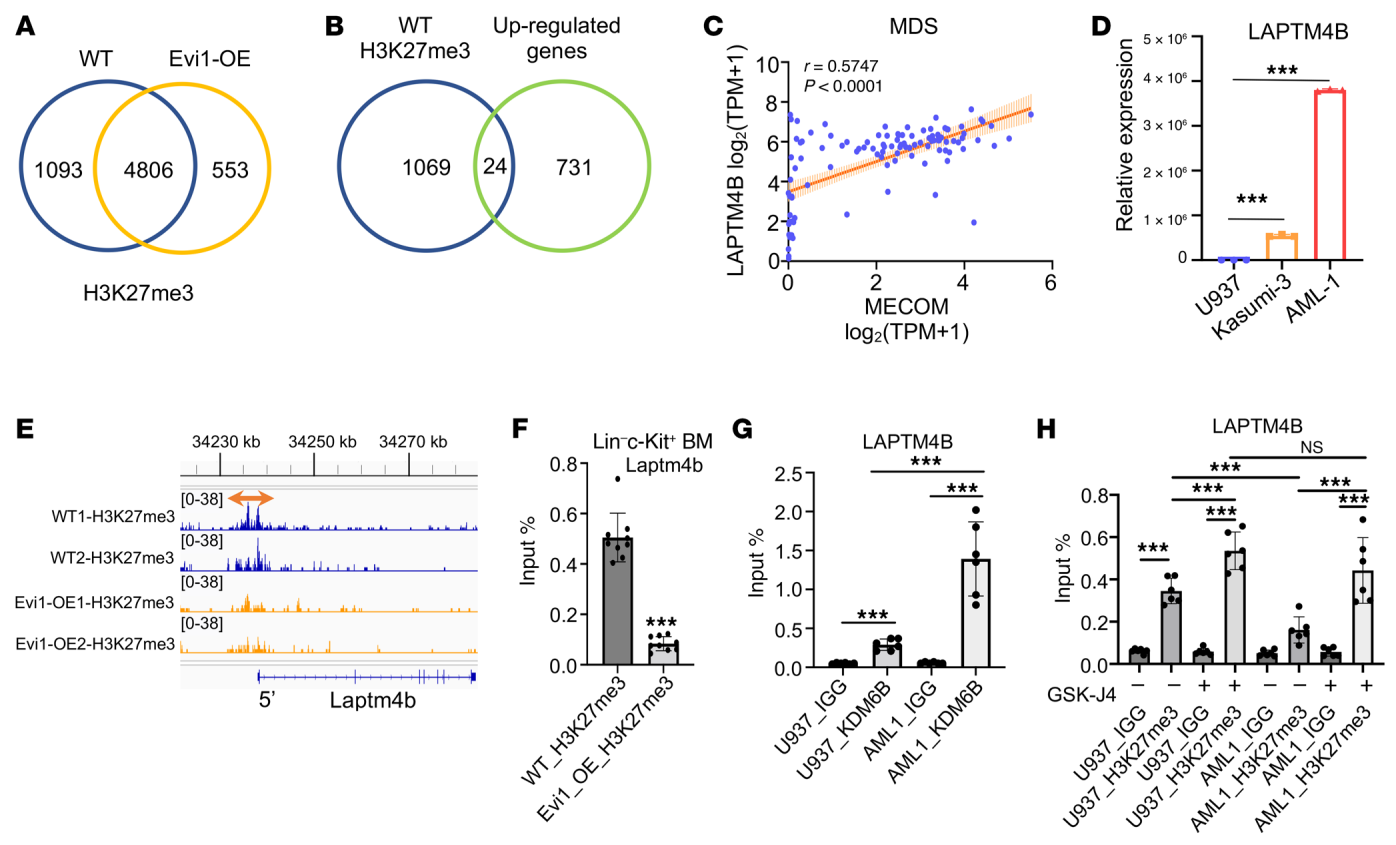
Laptm4b is identified as a functional mediator of Evi1 through Kdm6b-mediated histone demethylation. (**A**) Venn diagram showing the significantly H3K27me3-enriched genes in Lin^–^c-Kit^+^ cells from WT mice but not in Evi1-OE mice identified by CUT&RUN-seq analysis. (**B**) Venn diagram showing the overlap between H3K27me3-enriched genes in Lin^–^c-Kit^+^ cells from WT mice and the genes significantly upregulated in Lin^–^c-Kit^+^ cells from Evi1-OE mice. (**C**) Correlation between *EVI1* (*MECOM* in the database) and *LAPTM4B* in MDS patients (GEO GSE114922). (**D**) RT-qPCR analysis for transcription level of LAPTM4B in cell lines as indicated. (**E**) IGV peak visualization of H3K27me3 on the promoter of *Laptm4b* in WT or Evi1-overexpressing Lin^–^c-Kit^+^ mouse BM cells by CUT&RUN-seq analysis. (**F**) ChIP-qPCR analysis indicates that Evi1 overexpression significantly inhibits H3K27me3 enrichment at the promoter region of *Laptm4b* in Lin^–^c-Kit^+^ mouse BM cells. *n* = 3 for each group. (**G**) ChIP-qPCR analysis indicates that KDM6B directly binds to the promoter region of *LAPTM4B* in both U937 and AML1 cells. *n* = 2 for each group. (**H**) ChIP-qPCR analysis showing the effect of GSK-J4 treatment on H3K27me3 enrichment at the promoter region of *LAPTM4B* in U937 and AML1 cells. *n* = 2 for each group. In **C**, *P* value was calculated by Spearman’s *r* correlation. In **D** and **F**–**H**, data are represented as mean ± SD, ordinary 1-way ANOVA with Dunnett’s multiple-comparison test. Data are representative of at least 2 independent experiments. ***P < 0.001.

**Figure 7:**
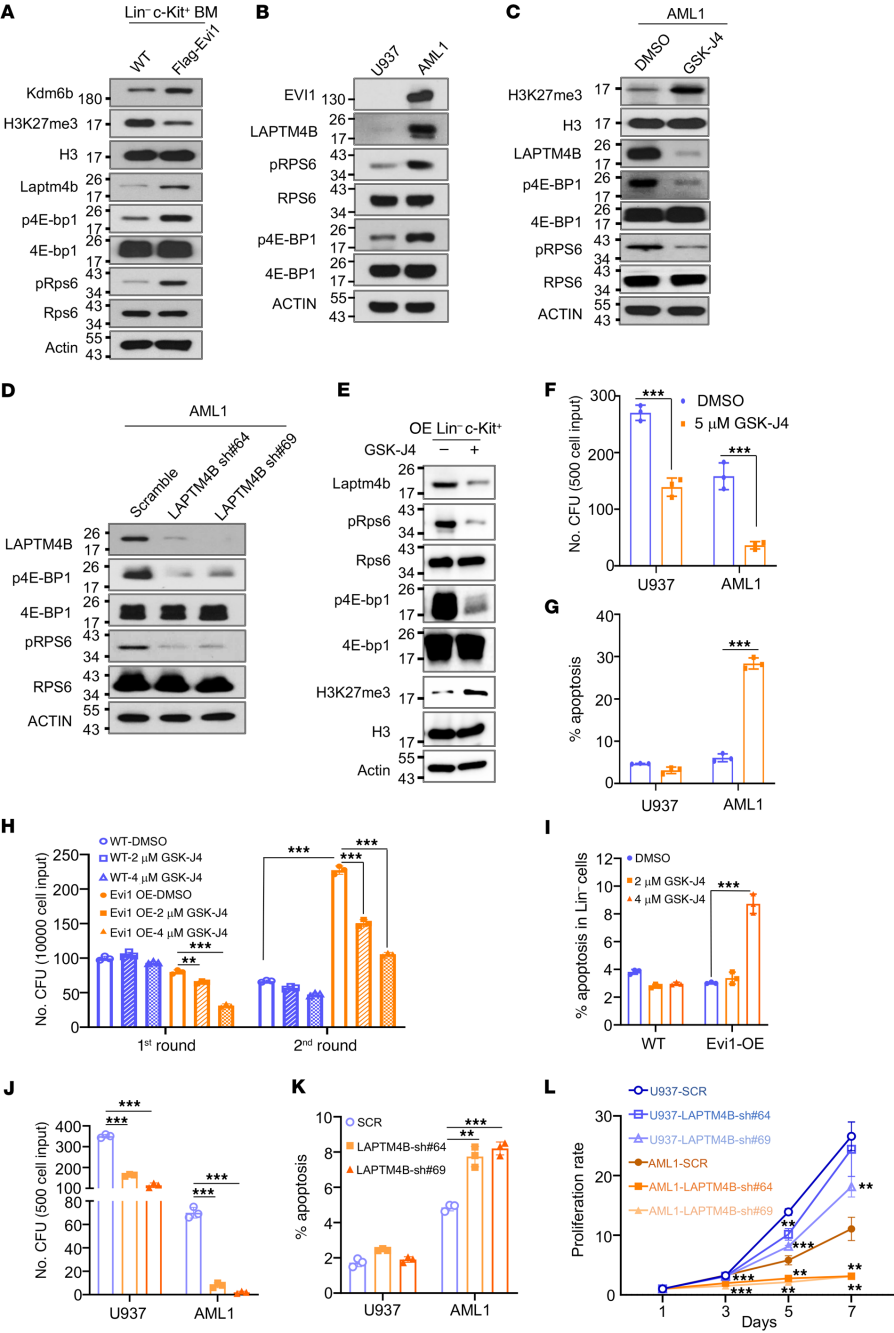
Evi1 overexpression activates mTOR pathway through Laptm4b. (**A**) WB analysis showing the expression level of indicated proteins in WT and Evi1-overexpressing Lin^–^c-Kit^+^ mouse BM cells. (**B**) WB analysis for the indicated proteins in U937 and AML1 cells. (**C**) WB analysis showing the effect of KDM6B inhibition on protein abundance as indicated. (**D**) WB analysis showing the effect of LAPTM4B knockdown on mTOR signaling in AML1 cells. (**E**) WB analysis showing the effect of KDM6B inhibition on protein abundance as indicated in Evi1-overexpressing Lin^–^c-Kit^+^ mouse BM cells. (**F**) Colony-forming assay of U937 and AML1 cells in methylcellulose cultures in the presence or absence of 5 μM GSK-J4. *n* = 3 for each group. (**G**) U937 and AML1 cells were treated with 5 μM GSK-J4 for 24 hours followed by flow cytometry analysis for apoptosis. *n* = 3 for each group. (**H**) Quantification of colony number of WT and Evi1-overexpressing BM cells cultured in methylcellulose with DMSO or GSK-J4. *n* = 3 for each group. (**I**) Frequency of apoptosis in Lin^–^ cells from colony-forming assay. *n* = 3 for each group. (**J** and **K**) Bar plots showing the effect of LAPTM4B knockdown on colony-forming ability (**J**) and cell apoptosis (**K**) of U937 and AML1 cells. *n* = 3 for each group. (**L**) Growth curve of U937 and AML1 cells transduced with LAPTM4B shRNAs or scramble. *n* = 3 for each group. Data are representative of at least 2 independent experiments. All bar graph data represent mean ± SD, and *P* values were determined by multiple *t* tests. **P < 0.01, ***P < 0.001.

To further elucidate the functional implications of Evi1, we performed integrative analysis using CUT&RUN and RNA-Seq profiles of Lin^–^c-Kit^+^ BM cells from WT and Evi1-overexpressing mice. We identified 1,093 genes with significantly higher peaks of H3K27me3 binding at their promoter region in WT Lin^–^c-Kit^+^ cells compared with Evi1-OE cells (Figure 6A). Among these genes, 24 genes, including *Laptm4b*, exhibited significantly higher expression levels (>2-fold) in Evi1-OE Lin^–^c-Kit^+^ cells compared with WT cells (Figure 6B and Supplemental Table 3). GO analysis of these 24 genes revealed that the lysosome pathway, in which *Laptm4b* is involved, was the top-ranked pathway associated with the DEGs (Supplemental Figure 5A). Analysis of a set of public data from patients with MDS (GEO GSE114922) showed a significantly positive correlation between *LAPTM4B* and *EVI1* expression (Figure 6C). Moreover, we observed higher expression of *LAPTM4B* in EVI1^hi^ Kasumi-3 and AML1 cells compared with EVI1^lo^ U937 cells (Figure 6D). Additionally, H3K27me3 CUT&RUN-seq analysis indicated that Evi1 overexpression led to a significant decrease in H3K27me3 enrichment at the promoter region of *LAPTM4B*, which was further verified by ChIP-qPCR analysis (Figure 6, E and F). Subsequently, we investigated the direct binding of KDM6B to the *LAPTM4B* promoter. Figure 6G demonstrates a significant enrichment of KDM6B at the promoter of *LAPTM4B* in both U937 and AML1 cells. Notably, the enrichment of KDM6B at the *LAPTM4B* promoter was higher in EVI1^hi^ AML1 cells compared with EVI1^lo^ U937 cells (Figure 6G). Consistently, we observed that the enrichment of H3K27me3 at the *LAPTM4B* promoter was higher in U937 cells than in AML1 cells, and these enrichments were more responsive to KDM6B inhibitor (GSK-J4) induction in AML1 cells compared with U937 cells (Figure 6H). Based on these findings, we hypothesized that Evi1 positively regulates *Laptm4b* through Kdm6b-mediated H3K27me3 demethylation at its promoter region. As anticipated, high expression of Evi1 led to a significant reduction in H3K27me3 levels in both Lin^–^c-Kit^+^ mouse BM cells and AML1 cells while upregulating protein levels of LAPTM4B (Figure 5G and Figure 7, A and B). Notably, these phenotypes were completely reversed upon inhibition of KDM6B in AML cells (Figure 7C). Previous research has indicated that LAPTM4B serves as an activator of mTOR signaling (44). Consistently, we observed that knockdown of LAPTM4B markedly inhibited mTOR signaling in AML1 cells (Figure 7D). Strikingly, high expression of Evi1 activated mTOR signaling in both Lin^–^c-Kit^+^ mouse BM cells and AML1 cells, and this EVI1-mediated activation of mTOR signaling could be reversed by KDM6B inhibition or LAPTM4B knockdown (Figure 7, A–E). In line with these findings, EVI1^hi^ AML1 cells exhibited a higher sensitivity to the KDM6B inhibitor compared with EVI1^lo^ U937 cells (Figure 7, F and G). Furthermore, GSK-J4 inhibited the colony-forming capacity and induced apoptosis of EVI1-overexpressing HSPCs, but it had a slight effect on WT HSPCs (Figure 7, H and I, and Supplemental Figure 5B).

Similarly, AML1 cells exhibited greater sensitivity to LAPTM4B knockdown–induced cell proliferation arrest and cell apoptosis compared with U937 cells (Figure 7, J–L). Collectively, these results suggest that EVI1 facilitates the expression of LAPTM4B, thereby activating the mTOR signaling pathway, likely through KDM6B-mediated H3K27me3 demethylation at the promoter region of *LAPTM4B* in both Lin^–^c-Kit^+^ mouse BM cells and AML cells.

### Suppression of Laptm4b partially rescues abnormal hematopoiesis induced by Evi1 overexpression.

**Figure 8:**
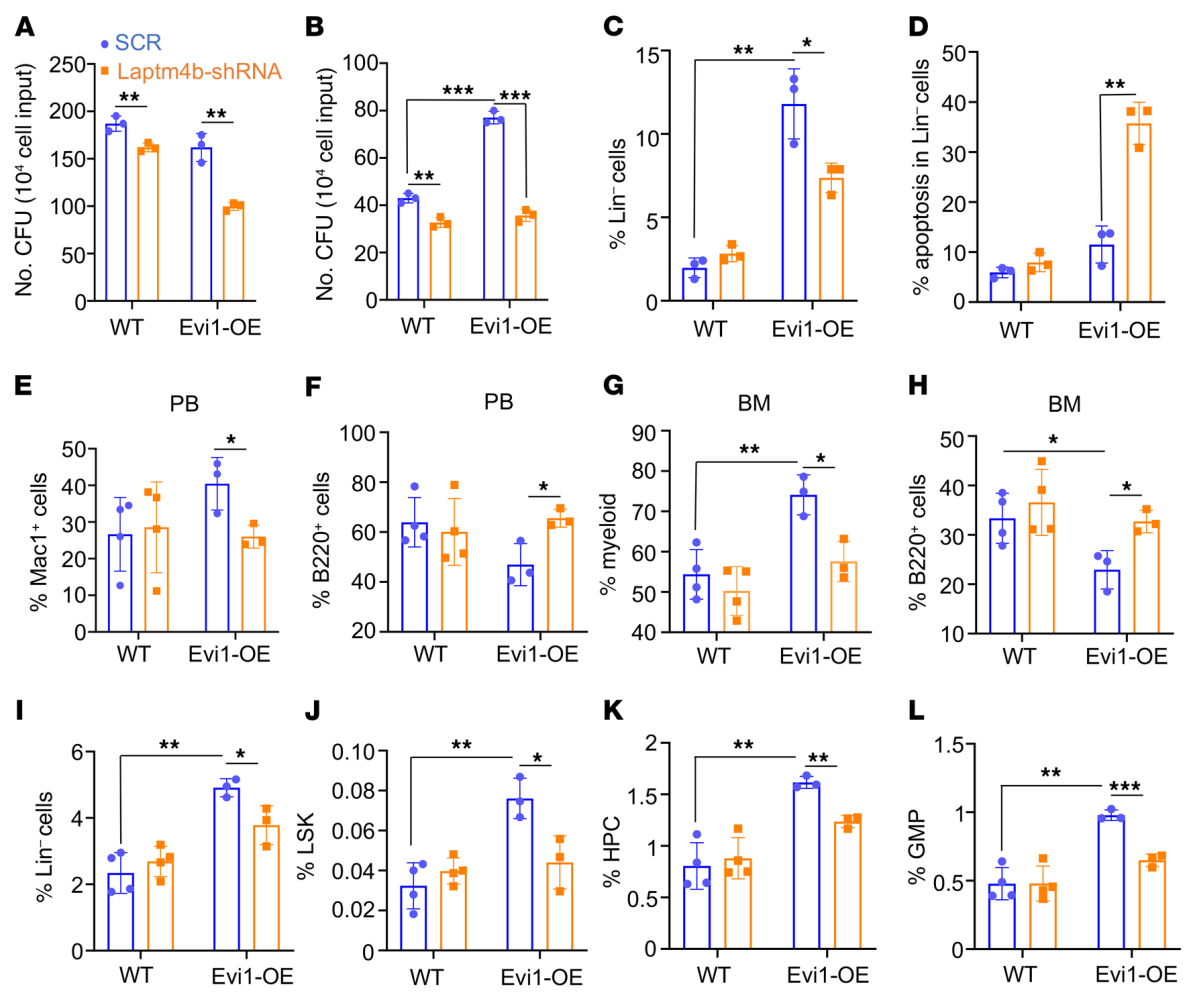
Suppression of Laptm4b partially rescues hematopoietic disorders caused by Evi1 overexpression. (**A** and **B**) Quantification of colony number for the first round (**A**) and second round (**B**) of the colony-forming assay using BM cells transduced with scramble and Laptm4b shRNA. (**C**) Frequency of Lin^–^ cells in bone cells from colony-forming assay. (**D**) Frequency of apoptosis in Lin^–^ BM cells from colony-forming assay. In **A**–**D**, *n* = 3 for each group. (**E** and **F**) Frequency of Mac1^+^ cells (**E**) and B220^+^ cells (**F**) originating from WT or Evi1-overexpressing donor cells in PB 6 weeks after transplantation. (**G** and **H**) Frequency of myeloid (Mac1^+^Gr1^+^) (**G**) and B220^+^ cells (**H**) originating from WT or Evi1-overexpressing donor cells in BM 6 weeks after transplantation. (**I**–**L**) Frequency of Lin^–^ cells (**I**), LSKs (**J**), HPCs (**K**), and GMPs (**L**) originating from WT or Evi1-overexpressing donor cells in the BM 6 weeks after transplantation. In **E**–**L**, *n* = 4 for the WT groups and *n* = 3 for the Evi1-OE groups. Data are representative of at least 2 independent experiments. All bar graph data represent mean ± SD, and *P* values were determined by multiple *t* tests. *P < 0.05, **P < 0.01, ***P < 0.001.

Given that Evi1 overexpression promoted aberrant hematopoiesis in recipient mice after BM transplantation (Figure 3L), we next sought to evaluate the impact of Laptm4b knockdown on Evi1-induced abnormal hematopoiesis. To achieve this, we knocked down Laptm4b expression by Laptm4b-specific shRNAs (multiple shRNAs in a single vector system) (45) in Lin^–^c-Kit^+^ BM cells from WT and Evi1-OE mice (Supplemental Figure 5L). Lin^–^c-Kit^+^ hematopoietic cells from Evi1-OE mice exhibited greatly impaired clonogenic capacity after knockdown of Laptm4b in methylcellulose colony assays (Figure 8, A and B, and Supplemental Figure 5, C–F). Moreover, the frequency of expanded Lin^–^ progenitor cells was significantly decreased in Evi1-OE cells with Laptm4b knockdown, which was likely a consequence of Laptm4b knockdown–induced apoptosis (Figure 8, C and D, and Supplemental Figure 5K).

Next, we examined whether Laptm4b knockdown rescued the abnormal hematopoiesis induced by Evi1 overexpression in vivo. Lin^–^c-Kit^+^ BM cells from WT and Evi1-OE mice were infected with retrovirus expressing scramble or Laptm4b-specific shRNAs. These infected cells (CD45.2^+^) were mixed with helper cells (CD45.2^+^CD45.1^+^) and injected into lethally irradiated CD45.1^+^ recipient mice. Six weeks after transplantation, we noticed that the frequency of donor cells from each group was comparable in PB and BM (Supplemental Figure 5, G–J). Interestingly, we observed that Laptm4b knockdown significantly reversed Evi1-OE–enhanced myelopoiesis and inhibition of B cell differentiation (Figure 8, E–H). Laptm4b knockdown partially reversed the expansion of Evi1-OE Lin^–^ cells, LSKs, HPCs, and GMPs (Figure 8, I–L). Together, these data indicate that suppression of Laptm4b ameliorates clonal hematopoiesis associated with increased levels of Evi1 expression in vivo.

## Discussion

One important approach to gaining insights into the role of EVI1 in myeloid malignancies is the use of mouse models with *EVI1* overexpression. Here, we showed that a newly established EVI1-transgenic mouse model allows for the conditional activation of *Evi1* expression at a low level in HSPCs. In contrast to other Evi1-transgenic mouse models that developed AML (13, 21), our model demonstrated that *EVI1* overexpression leads to the development of MDS/MPN–like disease, characterized by dysregulated hematopoiesis, anemia, thrombocytopenia, and expansion of myeloid progenitor cells, which is consistent with the phenotypes being observed in EVI1^hi^ MDS and MDS/MPN patients due to inv(3)/t(3;3) rearrangements (17). Our model displays the phenotypes, similar to other mouse models of MDS/MPN (46, 47). Unlike in AML models, in which mice with secondary transplantation typically exhibit a more rapid development of the disease, our observation revealed a different pattern for Evi1-induced MDS/MPN. Through transplantation, the Evi1-induced MDS/MPN phenotype can be transferred to recipient mice; however, the onset of the disease was significantly delayed in comparison with the primary mice.

The difference between our Evi1-transgenic mouse model and other Evi1-transgenic models (13, 21) might be partially attributed to the varying levels of EVI1 expression in HSPCs across different mouse models. Our investigations showed that the exogenous Evi1 expression in this model is approximately 2–3 times higher than the endogenous Evi1 expression in LSKs. It is worth highlighting that about 27% of MDS patients with inv(3)/t(3;3) rearrangements progress to AML (48). This observation implies that additional acquired mutations, such as RAS mutations, are necessary for the transformation of EVI1^hi^ MDS into AML. Hence, our Evi1-transgenic model presents a unique opportunity to explore the role of EVI1 during the early stages of MDS, MDS/MPN, or AML diseases characterized by EVI1 overexpression.

Intriguingly, we found that modest changes in Evi1 overexpression in HSPCs are sufficient to induce MDS/MPN in mice in vivo. Our observations revealed that Evi1 overexpression actively drives cell cycling in LT-HSCs, leading to enhanced differentiation into committed myeloid progenitor cells and simultaneous inhibition of differentiation into committed lymphoid progenitor cells. Consequently, we observed an expansion of MPP2 and MPP3 committed myeloid progenitors, along with a reduction in MPP4 lymphoid progenitor cells. However, this increase in EVI1 expression also resulted in a decrease in the number of LT-HSCs. Consistent with these findings, competitive repopulation assays demonstrated a significant expansion of myeloid progenitors overexpressing Evi1, contrasting with the behavior of HSCs, when compared with their WT counterparts. Interestingly, the recipient mice that received Evi1-OE BM cells exhibited milder phenotypes compared with primary Evi1-OE mice, suggesting that Evi1 overexpression may also induce dysfunction of niche cells in the BM microenvironment, which may contribute to Evi1-induced aberrant hematopoiesis in this mouse model. These findings propose that the upregulation of EVI1 may play a pivotal role in the initiation of clonal hematopoiesis in patients with MDS, MDS/MPN, or AML with EVI1 overexpression during the early stages of the disease.

Many downstream targets and pathways of EVI1 in hematopoietic cells have been identified so far (24). Evi1 can regulate its target genes by either activating or inhibiting them, depending on the specific context of its function. However, numerous studies have been conducted using cell lines to investigate these molecular functions of transcription factors. Since these functions are cell context dependent, it is necessary to unravel the specific activities of EVI1 in primary cells. The global Evi1 binding sites in primary HSPCs have not been reported. By CUT&RUN assay, we have identified thousands of genes that contain the EVI1 binding sites in their regulatory regions. By integrative analysis of EVI1-induced gene expression changes and its global binding sites, we found that hundreds of genes were upregulated or downregulated when they were directly bound by Evi1, suggesting that they are directly regulated by Evi1. GO analysis revealed that EVI1 direct target genes are involved in a variety of biological processes, such as myeloid differentiation, RNA splicing, and protein folding, which may mediate the function of Evi1 in HSPCs. Numerous studies have reported the diverse functions of EVI1 in hematopoiesis. EVI1 transcriptionally represses the expression of C/EBPα, RUNX1, GATA1, and PU.1, leading to the blockage of lineage differentiation (49–52). EVI1 has also been reported to act as a transcriptional repressor by recruiting the DNA methyltransferases DNMT3A and DNMT3B to induce aberrant DNA methylation at the promoter regions of its target genes. Additionally, EVI1 interacts with a subset of histone deacetylases and histone methyltransferases, such as SUV39H1 and the polycomb repressor complex 2 (PRC2), to induce a condensed chromatin structure (23, 53–55). Conversely, we observed that Evi1 overexpression led to increased protein levels of Kdm6b and a decreased protein level of H3K27me3 in both mouse primary cells and human EVI1^hi^ AML cell lines. Consistently, by integrative analysis of Evi1-induced gene expression changes and global H3K27me3 binding sites, we observed that a number of genes, including *Laptm4b*, that were upregulated by Evi1 exhibited a significantly decreased H3K27me3 enrichment in their promoter region, indicating that these genes are regulated by Kdm6b-mediated H3K27me3 demethylation.

KDM6B is overexpressed in a variety of blood disorders, including myelodysplastic syndromes, M5 acute myeloid leukemia, Hodgkin’s lymphoma, multiple myeloma, and T cell acute lymphoblastic leukemia (56–60). KDM6B exhibits opposing roles in leukemia development. KDM6B acts as a tumor suppressor in M2/M3 AML, where its downregulation blocks differentiation and is associated with poor prognosis (61). Interestingly, constitutive overexpression of Kdm6b in the hematopoietic system disrupts hematopoiesis and leads to pathologies akin to human MDS (58). Another study revealed that the loss of Kdm6b impaired HSC function through the upregulation of the AP-1 transcription factor complex (Fos and Jun) during the HSC stress response (41). Our study, however, showed that the AP-1 transcription factor complex (Fos and Jun) is upregulated in Evi1-OE HSPCs, which is consistent with the findings previously reported (30). It is possible that Evi1 overexpression overrides the effect of Kdm6b upregulation on these two genes in HSPCs. Our results suggest an oncogenic role of Kdm6b-mediated H3K27me3 downregulation in EVI1-induced myeloid malignancies.

The mammalian target of rapamycin (mTOR) pathway plays a crucial role in sensing cellular energy levels and regulating protein and lipid synthesis, thereby maintaining normal hematopoiesis (62, 63). In AML patients, uncontrolled malignant cell growth often arises from disruption in intracellular signaling caused by mutations or aberrant external signaling, and aberrant upregulation of the mTOR pathway has been observed in patients with AML (64–66). However, the mechanisms underlying the constitutive activation of mTOR signaling in AML are not completely understood. We discovered that Evi1 specifically activates the transcription of Laptm4b by Kdm6b-mediated H3K27me3 demethylation at its promoter region. This activation leads to the induction of the mTOR signaling pathway in both mouse primary HSPCs and human AML cell lines. Consistent with this, a previously published study suggests that Laptm4b activates mTOR signaling by recruiting the LAT1-4F2hc Leu transporter to lysosomes (44). We found that EVI1^hi^ AML cells displayed increased sensitivity to KDM6B inhibitor and LAPTM4B knockdown. Importantly, we observed a positive correlation between the expression of LAPTM4B and EVI1 in patients with MDS. Indeed, Laptm4b knockdown partially reversed Evi1-OE–induced abnormal hematopoiesis and expansion of HSPCs. Overall, these findings suggest that pharmacologic approaches to interrupt the Evi1/Kdm6b/H3K27me3/Laptm4b/mTOR signaling axis may serve as important strategies to treat EVI1^hi^ hematopoietic malignancies.

## Methods

### Sex as a biological variable.

In all mouse studies, both male and female mice were used. Sex was not considered as a biological variable in the statistical analyses.

### Mice.

A conditional Evi1-transgenic model was developed using the TARGATT system in the B6 background strain. The Transgenic and Targeted Mutagenesis Laboratory at Northwestern University (Chicago, Illinois, USA) assisted in generating this mouse model. The pBT346 vector and TARGATT kit, which includes the TARGATT integrase (Ф31) (the specific integrase used in the mouse model), were obtained from Applied Stem Cells. The mouse Evi1 gene was subcloned from the pGCDNsam-mEvi1-EGF vector, and a FLAG epitope was introduced at the N-terminus of Evi1. In the conditional Evi1-transgenic mice, a knockin Evi1 transgene (Tg) with an additional exon containing a stop codon flanked by LoxP (LoxP-stop-LoxP [LSL]) recombination sequences in the correct sequence orientation was specifically integrated into the Rosa26 locus to ensure a stable transgene expression upon inducing the expression of Cre recombinase. Tg (LSL-Evi1) mice were then crossed with Mx1-Cre transgenic mice to obtain Mx1-Cre Tg (LSL-Evi1) mice. Overexpression of the Evi1 transgene was induced by administration of 2 intraperitoneal injections of 10 mg poly(I:C) per gram of body weight every other day. CD45.1 C57BL/6 (B6) mice used as recipients in transplantation assay were purchased from The Jackson Laboratory. All experimental procedures were conducted following protocols approved by the Institutional Animal Care and Use Committee of the University of Florida.

### Antibodies.

All information about the antibodies applied in this study is listed in Supplemental Table 1.

### Biopsy.

Decalcified and fixed BM core biopsy specimens were prepared. Histologic sections of biopsy and aspirate clot specimens were stained with hematoxylin and eosin. Peripheral blood smears and BM aspirate smears were stained with May-Grünwald-Giemsa.

### Peripheral blood cell counting.

Peripheral blood was collected from the tail vein into tubes containing EDTA (Sarstedt Inc.). The whole-blood counts, including white blood cell, RBC, and platelet counts and hemoglobin level, were determined using a Hemavet 950FS (Drew Scientific Inc.).

### Cell culture.

All cell lines were purchased from American Type Culture Collection or DSMZ. Primary mouse BM cells were cultured in Iscove’s modification of DMEM (Corning 10-016-CV) with 20% fetal bovine serum (FBS), 1% penicillin-streptomycin (PS), 10 ng/mL IL-3, 10 ng/mL IL-6, 100 ng/mL SCF, and 0.05 mM 2-mercaptoethanol. Erythroid myeloid lymphoid (EML) cells were cultured in IMDM supplemented with 20% FBS, 4 μM l-glutamine, 1.5 g/L sodium bicarbonate, and 200 ng/mL mouse SCF. U937 cells and Kasumi-3 cells were incubated in RPMI 1640 media supplemented with 10% FBS and 1% PS. AML1 cells were cultured in RPMI 1640 medium supplemented with 10 ng/mL human-GM-CSF, 10% FBS, and 1% PS. For GSK-J4 treatment, 5 μM GSK-J4 (Selleck Chemicals LLC, catalog S7070) was added to the medium to treat the cells as indicated.

### Virus production and infection.

For KDM6B and LAPTM4B shRNA lentivirus production, the shRNA plasmids together with 2 packaging plasmids, pMDG.2 and Δ8.91 (a gift from Adrian J. Thrasher), were transfected into HEK293T cells by polyethylenimine. Starting 24 hours after transfection, the supernatant medium containing virus was collected every 12 hours, 4 times. The cells were mixed with a virus medium consisting of 4 μg/mL Polybrene followed by spinoculation at 300 *g* for 3 hours at 32°C. The spinoculation was repeated the following day. After 48 hours of spinoculation, 2 μg/mL puromycin was added to select positively infected cells.

Laptm4b knockdown in mouse BM cells was performed as previously described (45). The targeting sequences were designed using BLOCK-iT RNAi Designer (Invitrogen). These oligonucleotide cassettes were assembled into a retroviral shuttle vector using the FAMSi system, and subsequently cloned into the retroviral backbone vector pSiEB (provided by Tong-chuan He, Department of Orthopaedic Surgery and Rehabilitation Medicine, University of Chicago, Chicago Illinois, USA). Retroviral vector–mediated transduction was performed as described in our previous study (67).

### Western blot.

Freshly collected cell pellets were lysed and boiled in 2% SDS loading buffer. SDS-PAGE was performed to separate protein samples. The proteins were then electroblotted onto polyvinylidene difluoride membranes (Thermo Fisher Scientific). The membranes were blocked with 5% milk powder in PBS and incubated with specific antibodies against Evi1 (Invitrogen), FLAG (MilliporeSigma), or actin (Invitrogen). Horseradish peroxidase–conjugated secondary antibodies and substrate (MilliporeSigma) were used for chemiluminescence analysis.

### Lin^–^c-Kit^+^ BM cell isolation.

BM cells were harvested from the femora and tibiae of moribund Evi1-OE and control mice. After lysing of erythrocytes, BM-derived mononuclear cells were washed with PBS containing 0.5% bovine serum albumin and 2 mM EDTA (Gibco). After depletion of the Lin^+^ BM cells according to a previously established protocol (67), the Lin^–^ BM cells were resuspended in washing buffer and incubated with mouse monoclonal CD117 microbeads (Miltenyi Biotec 130-091-224) for 15 minutes at 4°C. c-Kit^+^ bone marrow cells were enriched by magnetic-activated cell sorting (MACS) and used for RNA isolation and assessment of purity.

### Flow cytometry analysis.

Single-cell suspensions from BM, spleen, peripheral blood, and thymus were stained with fluorochrome-conjugated antibodies (BD Biosciences). Flow cytometric analysis of HSCs, subsets of HPCs, and mature cell populations has been previously described (29). Cell cycle analysis with pyronin Y and Hoechst 33342 or Ki67 and 4′6-diamidino-2-phenylindole (DAPI) staining was performed as previously described (29). Flow cytometry was performed using Cyan or CytoFLEX S flow cytometers (Beckman). All data were analyzed using FlowJo software (Tree Star Inc.).

### Colony-forming assay.

A total of 5 × 10^3^ BM cells isolated from Evi1-OE and WT mice 1 month after poly(I:C) induction were plated in duplicate in methylcellulose medium (MethoCult, StemCell Technologies) supplemented with mouse IL-3, IL-6, and SCF. Colonies were scored 7 days after plating. Serial replating was performed after scoring.

For colony-forming assay (CFA) analysis with human cell lines, including U937 and AML1 cells transduced with LAPTM4B shRNAs or scramble, 500 cells were plated in triplicate in human Methylcellulose Base Media (R&D Systems HSC002) and scored 10 days after plating. For the treatment assay, 500 cells were plated in triplicate in human Methylcellulose Base Media with or without 5 μM GSK-J4.

For CFA analysis with BM cells treated with GSK-J4, 1 × 10^4^ cells were plated in methylcellulose medium (MethoCult) supplemented with mouse IL-3, IL-6, SCF, and indicated dosage of GSK-J4. Colonies were scored 7 days after plating. Serial replating was performed after scoring.

For CFA analysis with Lin^–^c-Kit^+^ BM cells, the isolated cells were cultured overnight and then transduced with scramble and Laptm4b shRNA through spinoculation. After selection with blasticidin for 48 hours, 1 × 10^4^ cells were plated in methylcellulose medium (MethoCult) supplemented with mouse IL-3, IL-6, SCF, and blasticidin. Colonies were scored 7 days after plating. Serial replating was performed after scoring.

### Transplantation.

For competitive repopulation assay, the CD45.1^+^ recipients received lethal irradiation followed by retro-orbital injection with 100 μL cell suspension including 1 × 10^6^ BM donor cells from Mx1-Cre Tg (LSL-Evi1) or Tg (LSL-Evi1) mice (CD45.2^+^) and 1 × 10^6^ CD45.1^+^CD45.2^+^ competitor cells. Four weeks later, these mice were injected with 3 doses of poly(I:C) every other day. The ratio of donor to competitor cells in peripheral blood was analyzed monthly by flow cytometry for 4 months. BM cells were analyzed by flow cytometry after 4 months of poly(I:C) injection.

For the BM transplantation (BMT) mouse model in Figure 3, BM cells from Mx1-Cre Tg (LSL-Evi1) or Tg (LSL-Evi1) mice injected with 2 doses of poly(I:C) were transplanted into lethally irradiated CD45.1^+^ mice. The peripheral blood was harvested every month for complete blood count analysis for 5 months. BM cells were analyzed at the fifth month after transplantation. For the BMT mouse model in Supplemental Figure 3, BM cells from Mx1-Cre Tg (LSL-Evi1) or Tg (LSL-Evi1) mice without poly(I:C) injection were transplanted into lethally irradiated Ly5.1 mice. Poly(I:C) injection and the flow cytometry analysis of peripheral blood were performed as described above.

For the Evi1-OE BMT mouse model in Figure 8, Lin^–^c-Kit^+^ BM cells from Mx1-Cre Tg (LSL-Evi1) or Tg (LSL-Evi1) mice injected with 2 doses of poly(I:C) were isolated and transduced with scramble and Laptm4b shRNA through spinoculation. After selection with blasticidin, transduced cells together with 1 × 10^6^ CD45.1^+^CD45.2^+^ helper cells were transplanted into lethally irradiated CD45.1^+^ mice. After 6 weeks, peripheral blood was harvested, and the mice were sacrificed and analyzed by flow cytometry.

### Quantitative real-time PCR.

Total RNA from the indicated cells was isolated using TRIzol (Invitrogen) and phenol-chloroform. RT-qPCR was performed on an Applied Biosystems 7500 thermocycler using the primer sequences listed in Supplemental Table 2 and analyzed via the ΔΔCt method.

### Chromatin immunoprecipitation.

Cells were cross-linked by 1% paraformaldehyde for 15 minutes at room temperature and terminated by 125 mM glycine. About 2 million cells were used for ChIP-qPCR analysis. The ChIP assay was performed as previously described (68). All the primer sequences for ChIP-qPCR analysis are listed in Supplemental Table 2.

### CUT&RUN-seq.

CUT&RUN assay (69) was performed with a commercially available kit according to the manufacturer’s instructions (pAG-MNase, EpiCypher catalog 15-1116). Briefly, about 10 ng of the purified CUT&RUN DNA was used for the preparation of multiplexed libraries with the NEB Ultra II DNA Library Prep Kit according to the manufacturer’s instructions (New England Biolabs E7103). Sequencing was conducted using an Illumina NextSeq 500 Sequencing System (available from the core facility of the University of North Carolina Pharmacology Department).

### CUT&RUN data analysis.

FASTQ files were mapped to the reference genome (mm10 for mouse genome) using bowtie2.3.5 (70). The nonprimary alignment, PCR duplicates, and blocklist regions were removed by Samtools (v1.9) (http://www.htslib.org/), Picard MarkDuplicates function (v2.20.4) (https://broadinstitute.github.io/picard/), and bedtools (v2.28.0) (https://bedtools.readthedocs.io/en/latest/), respectively. Peak calling was performed using MACS2 (macs2 callpeak -f BAMPE -g mm --keep-dup 1) (71). The distribution of peaks was calculated by the annotatePeak function of HOMER (Hypergeometric Optimization of Motif Enrichment) (https://bowtie-bio.sourceforge.net/bowtie2/index.shtml) (72). DeepTools (v3.3.0) was used to make bigwig files (--normalizeUsing RPKM), heatmaps, and averaged plotting of CUT&RUN signals (73). Genomic binding profiles were generated using the deepTools bamCompare functions.

### RNA sequencing.

Total RNAs from sorted LSKs or Lin^–^c-Kit^+^ cells were isolated as described above. The library was prepared by Library Construction Kit (Clontech). RNA sequencing (RNA-Seq) for LSKs was performed on an Illumina HiSeq 3000 system (Illumina Inc.) with 50 bp single-read mode by the Clinical Microarray Core at UCLA. RNA-Seq for Lin^–^c-Kit^+^ cells was performed on the Illumina NovaSeq system with paired-end 150 bp mode by the UF Health Cancer Center. The sequencing depth was 30 million reads per sample. RNA-Seq analysis for LSKs was performed using Partek Flow software (v10.0) (https://www.partek.com/partek-flow/). For RNA-Seq of Lin^–^c-Kit^+^ cells, quality check of raw reads and alignment by Picard and HISAT2 (https://daehwankimlab.github.io/hisat2/). Read count data were processed using the R edgeR package for filtering and normalization. GSEA was performed with GSEA v4.0.0 software, available from the Broad Institute (http://www.broad.mit.edu/gsea/; Massachusetts Institute of Technology, Cambridge, Massachusetts, USA).

### Statistics.

Results are presented as mean ± SD. Statistical significance was calculated with 2-tailed Student’s *t* test, ordinary 1-way ANOVA, or 2-way ANOVA with Dunnett’s multiple-comparison test using GraphPad Prism v8.0 software. Survival curves were compiled using Kaplan-Meier algorithms of GraphPad Prism, and significance was calculated by the log-rank (Mantel-Cox) test. Correlation was calculated according to Spearman’s statistical analysis by GraphPad Prism. *P* less than 0.05 was considered statistically significant.

### Study approval.

All the animal experiments were conducted under the approval of the University of Florida Institutional Animal Care and Use Committee.

### Data availability.

The sequencing data including RNA-Seq of LSKs, RNA-Seq of Lin^–^c-Kit^+^ cells, and CUT&RUN-seq of Lin^–^c-Kit^+^ cells were deposited in the GEO database (accession numbers GSE235441, GSE235440, and GSE235439, respectively). Values for all data points in graphs are reported in the Supporting Data Values file.

## Author contributions

ZQ conceived the project. ZQ and JL designed the experiments. QW, CY, YS, YL, LL, and CH performed in vivo experiments and interpreted the results. FY, CY, and JW performed molecular experiments and cell culture experiments and interpreted the results. YG performed CUT&RUN sequencing and analyzed the data. QW and CY performed RNA-Seq and analyzed the data. YZ and YH analyzed RNA-Seq data and CUT&RUN data. HN provided histologic analysis. ZQ, QW, CY, and FY contributed to the preparation of the manuscript. TH, ZH, WW, and GGW provided advice and new reagents/analytic tools. All authors provided a critical review of the manuscript.

## Supplementary Material

Supplemental data

Unedited blot and gel images

Supporting data values

## Version 1 12/16/2024

Electronic publication
