# Supplementary material for: Evi1 governs Kdm6b-mediated histone demethylation to regulate the Laptm4b-driven mTOR pathway in hematopoietic progenitor cells
Source: J Clin Invest. 2024 Dec 16;134(24):e173403. doi: 10.1172/JCI173403 (PMC11645144; doi:10.1172/JCI173403)
Supplement: Supplemental data [file jci-134-173403-s137.pdf]

Supplemental Figure 1

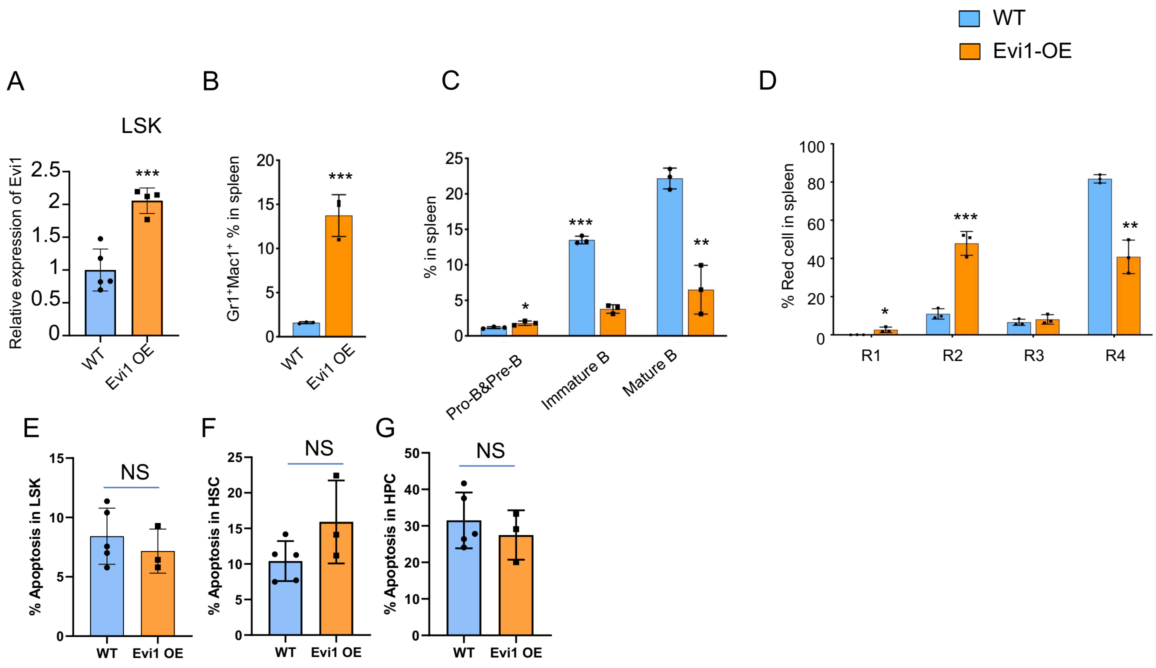

Supplemental Figure 1. Evi1 overexpression induces abnormal hematopoiesis in mice. (A) Relative expression of Evi1 in LSKs. n=5 for WT mice, n=4 for Evi1 OE mice. (**B**) - (**D**) Flow cytometric analysis of the frequency of Myeloid cells. **(B),** B cells **(C)** and erythroid in spleen**(D)**. n=3 for each group. (**E**) - (**G**) Flow cytometric analysis of apoptosis in LSKs, LT-HSCs and HPC. Data are representative of at least 2 independent experiments and represented as mean ± s.d., 2-tailed Student's t test. *p <0.05, **p < 0.01, ***p < 0.001.

Supplemental Figure. 2

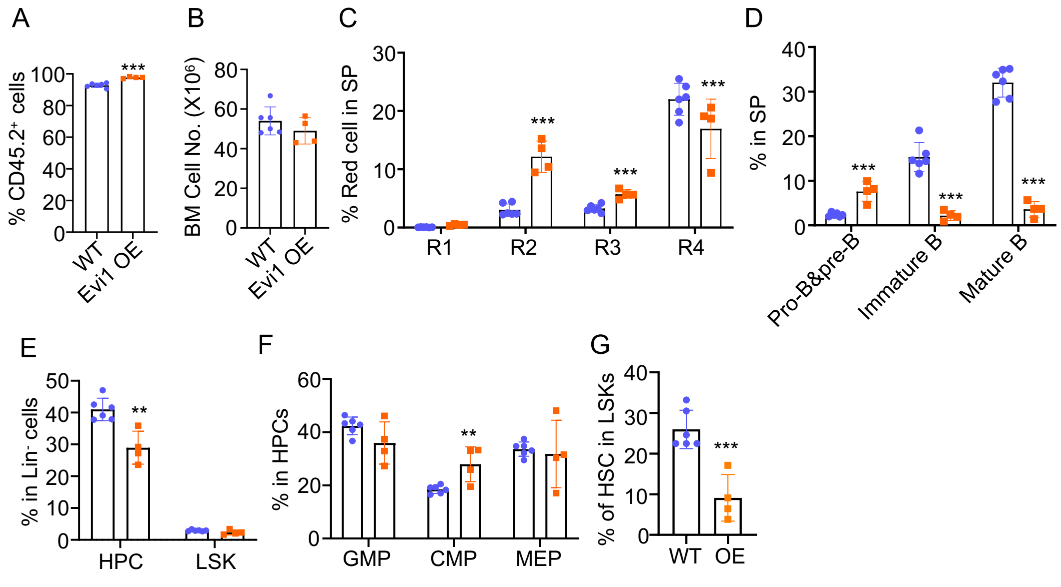

Supplemental Figure 2. Evi1 overexpression induced MDS/MPN-like disease in recipient mice. (A) Engraftment efficiency was confirmed in BM cells from the recipient mice transplanted with WT or Evi1-OE BM cells. **(B)** Total BM cells number from the recipient mice transplanted with WT or Evi1-OE BM cells. **(C) - (D)** The frequency of red cells**(C)** and B cells**(D)** in spleen from the recipient mice transplanted with WT or Evi1-OE BM cells. **(E) - (G)** The frequency of HSPC populations in BM from the recipient mice transplanted with WT or Evi1-OE BM cells. Data are representative of at least 2 independent experiments and represented as mean ± s.d., 2-tailed Student's t test**.** *p <0.05, **p < 0.01, and ***p < 0.001.

Supplemental Figure. 3

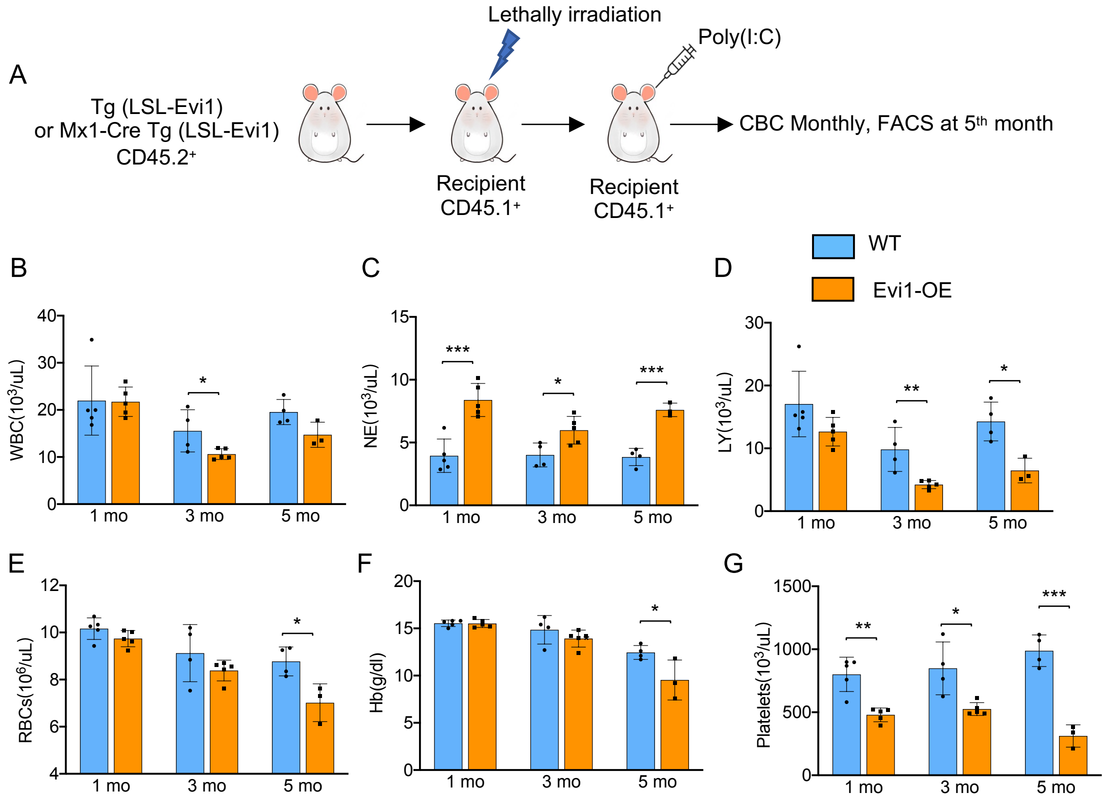

Supplemental Figure 3. Evi1 overexpression can induce MDS/MPN-like disease independent of microenvironment. **(A)** Schematic illustration of bone marrow transplantation. The activation of Evi1 was induced by poly(I:C) one-month post-transplantation. **(B) - (G)** Complete blood cell counts of the recipient mice from **A**. n=4-5 for WT group, n= 3-5 for Evi1-OE group. Data are representative of at least 2 independent experiments and represented as mean ± s.d., 2-tailed Student's t test. *p <0.05, **p < 0.01, and ***p < 0.001.

Supplemental Figure. 4

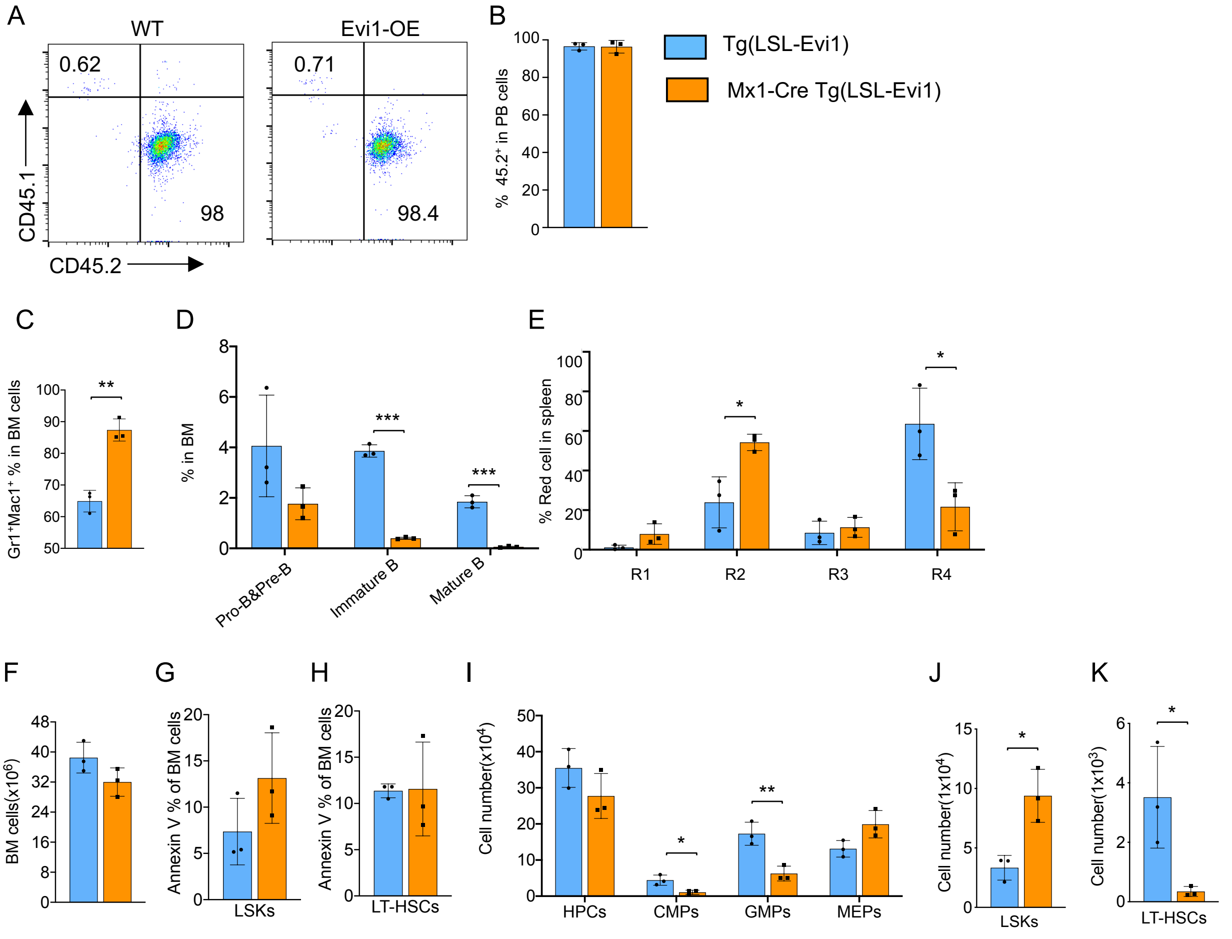

Supplemental Figure 4. Evi1 induced MDS/MPN-like disease independent of microenvironment. . (A) Representative flow cytometric plots showing engraftment efficiency in BM. **(B)** Percentage of transplanted cells in BM. **(C) - (D)** The frequency of myeloid cells **(C)** and B cells **(D)** in BM. **(E)** The frequency of red cells in spleen from the recipient mice transplanted with WT or Evi1-OE BM cells. **(F)** Total BM cell number from the recipient mice indicated in **Supplemental Figure 3A**. **(G) - (H)** The frequency of Annexin-V+ cells showing apoptosis level in LSKs and LT-HSCs. **(I)** Flow cytometric analysis of the frequency and the total number of HPCs and subsets of myeloid progenitors including CMPs, GMPs, and MEPs in the recipient mice. **(J) - (K)** Total cell number of LSK cells and LT-HSCs in the recipient mice. Data are representative of at least 2 independent experiments and represented as mean ± s.d., 2-tailed Student's t test. *p <0.05, **p < 0.01, and ***p < 0.001.

Supplemental Figure. 5

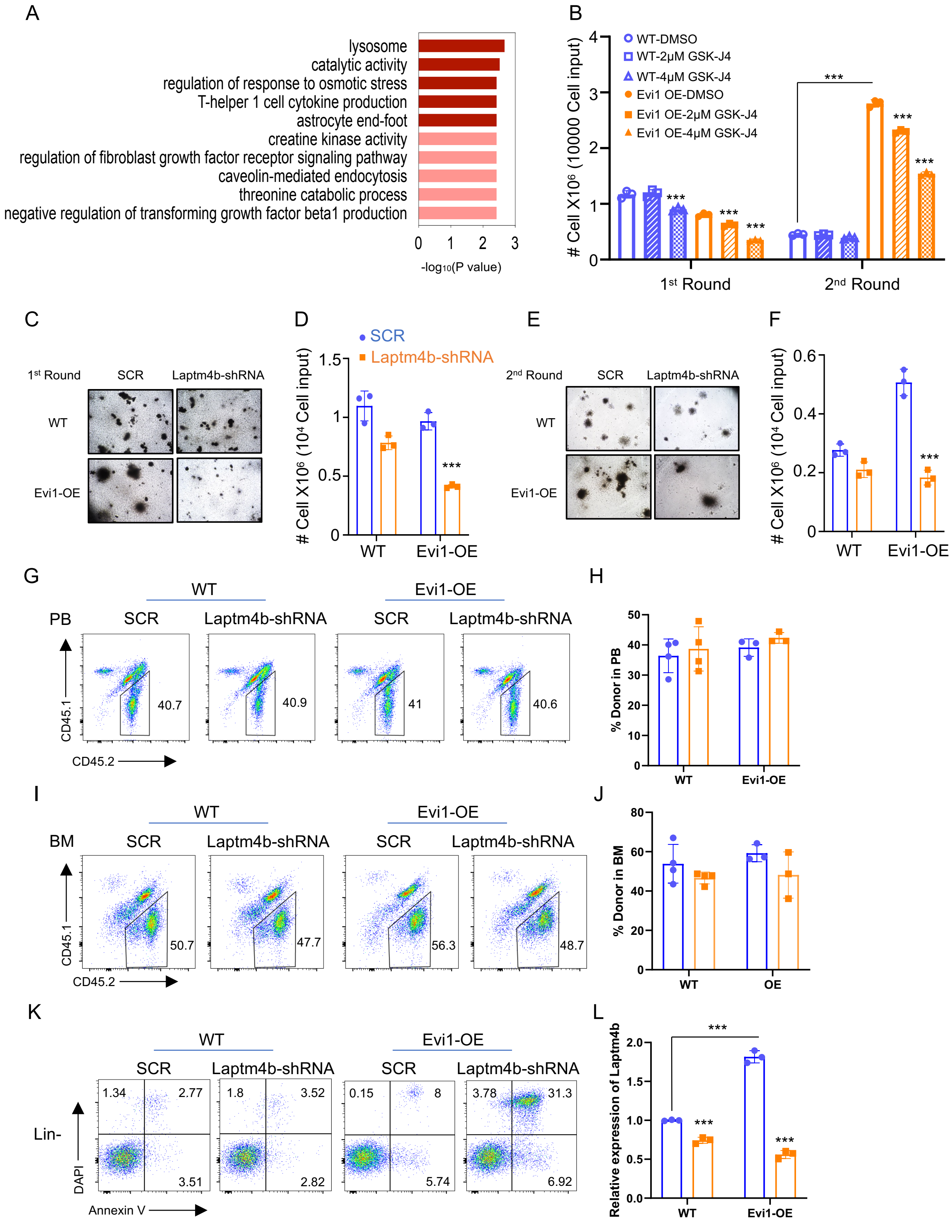

Supplemental Figure 5. Suppression of Laptm4b partially rescued hematopoietic disorders caused by Evi1 overexpression. (A) Gene ontology analysis of overlapped genes in Figure 6B. **(B)** The total cell number for the 1st round and 2nd round of colony forming assay in Figure 7H. **(C)-(D)** Representative images**(C)** and total cell number**(D)** for the 1st round of colony units of the BM cells transduced with Scramble and Laptm4b shRNA. **(E)-(F)** Representative images**(E)** and total cell number**(F)** for the 2nd round of colony units of the BM cells transduced with Scramble and Laptm4b shRNA. Photos were taken after 7 days of plating. **(G)** Representative flow cytometry plots showing the frequency of donor($CD45.2^+CD45.1^-$) in peripheral blood. **(H)** Quantification of the frequency of donor in PB. **(I)** Representative flow cytometry plots showing the frequency of donor in bone marrow. **(H)** Quantification of frequency of donor in BM. **(K)** Representative flow cytometry plots showing apoptosis gating in experiments performed in Figure 8G. **(L)** Relative expression of Laptm4b in Lin^- cells infected with lentivirus expressing Scramble or Laptm4b shRNA. Data are representative of at least 2 independent experiments. All bar graph data represent mean ± s.d., and P values were determined by multiple t tests. *p <0.05, **p < 0.01, and ***p < 0.001.

Supplemental Table 1: Antibodies

Antibodies used for FACS staining and immunostaining.	Supplier	Cat.
Streptavidin PE-Cy5	ebioscience	12-4317-82
Sca-1 PE	ebioscience	12-5981-83
Ckit APC-Cy7(efluor780)	ebioscience	47-1171-82
CD48 PE-Cy7	ebioscience	25-0481-80
CD150 APC	Biolegend	115910
CD16,32 PE-Cy7	ebioscience	25-0161-82
CD34 APC	ebioscience	50-0341-82
Ki67 FITC	ebioscience	11-5698-82
BrdU FITC	ebioscience	11-5071-42
CD45.1 PE	ebioscience	12-0453-83
CD45.2 FITC	ebioscience	11-0454-85
MAC APC	ebioscience	17-0112-82
Gr1 PE	ebioscience	12-5931-82
Gr1 APC-Cy7	ebioscience	47-5931-82
Ter119 APC	ebioscience	17-5921-82
CD71 PE	ebioscience	12-0711-82
F4/80 PE	ebioscience	12-4801-82
IgM APC	ebioscience	17-5790-82
B220 PE	ebioscience	12-0452-82
CD4 PE	ebioscience	12-0041-82
CD8a APC	ebioscience	17-0081-81
CD41 PE-Cy7	ebioscience	25-0411-82
CD49b biotin	ebioscience	13-5971-85
CD117 biotin	ebioscience	13-1171-85
CD3e Biotin	ebioscience	13-0033-86
CD45R (B220) Biotin	ebioscience	13-0452-86
Ly-6G (Gr-1) Biotin	ebioscience	13-5931-86
CD127 Biotin（IL7Ra）	ebioscience	13-1271-85
CD19 Biotin	ebioscience	13-0193-86
TER-119 Biotin	ebioscience	13-5921-85
Annexin V FITC	BD	556419
DAPI	Sigma	28718-90-3
Antibodies for Western blot, ChIP or IP	Supplier	Cat.
FLAG	Sigma	F3165
ACTIN	ptoteintech	23660-1-AP
H3K27me3	Invitrogen	MA5-11198

H3K27ac	Active Motif Inc	39134
H3K9me3	Active Motif Inc	39162
H3K4me1	Thermo fisher scientific	710795-20UG
H3K4me2	Active Motif Inc	39142
H3K4me3	Invitrogen	49-1005
Kdm6b	abcam	ab38113
H3	Invitrogen	PA5-16183
EVI1	Sigma	SAB4500736
p4E-bp1	Cell Signaling	2855
4E-bp1	Sigma	SAB4500736
pRps6	Cell Signaling	4856
Rps6	cell Signaling	2217
Mouse IgG	Sigma	NI03-100UG

Supplemental Table 2: Primers

Primer name	Sequence	Detail
Mx1-Cre-F	CTGCATTACCGGTCGATGCAAC	Detect Cre allele (301bp)
Mx1-Cre-R	GCATTGCTGTCACTTGGTCGTG	
R10	CTCTGCTGCCTCCTGGCTTCT	Detect the wildtype allele (311 bp)
R11	CGAGGCGGATACAAGCAATA	
PR425	GGTGATAGGTGGCAAGTGGTATTC	Detect the insertion (147 bp)
PR436	ATCAACTACCGCCACCTCGAC	
qPCR-mEvi1-F	TTA ATA TGA GTC ATG CCA ACC CG	qPCR primers for mouse Evi1
qPCR-mEvi1-R	GAC TAG CGG GTA TCA AAG GAG GC	
RT-human EVI1-F	CTCAGCATTTTCAATGGTTGA	qPCR primers for human EVI1
RT-human EVI1-R	TGTGACAGCATGTGTTTCTCC	
hKDM6B-RT-F	GGAGGCCACACGCTGCTAC	qPCR primers for human KDM6B
hKDM6B-RT-R	GCCAGTATGAAAGTTCCAGAGCTG	
m-Kdm6b-RT-F	TGAAGAACGTCAAGTCCATTGTG	qPCR primers for mouse Kdm6b
m-Kdm6b-RT-R	TCCCGCTGTACCTGACAGT	
qPCR human-LAPTM4B-F	GCCCGGAGCGATGAAGATG	qPCR primers for human LAPTM4B
qPCR human-LAPTM4B-R	CAACAGTACCACAGCATTGATGA	
qPCR mLaptm4b-F	GCTCCCTGGACTCGGTTCTA	qPCR primers for mouse Laptm4b
qPCRmLaptm4b-R	GCAGGACCACAGCATTGATGA	
mKdm6b-Promoter-F	atctccccggagaaaagagctggag	ChIP-qPCR primers to validate the binding site of Evi1 on the promoter of Kdm6b in mouse Lin$^-$ c-Kit$^+$ cells or EML cells
mKdm6b- Promoter-R	aagtaatcccagtcccgcttgggat	
hKDM6B-P1-F	TCAACTGCTTTGTGCATTTTATTTG	ChIP-qPCR primers to

hKDM6B-P1-R	AACGAAAGGCTCCAGAGATCAACAC	validate the binding site of Evi1 on the promoter of KDM6B in human cell lines
m-Laptm4b-5UTR-1-F	GTCCAGCAAGGAAGCATTTATT	ChIP-qPCR primers to validate the modified sites of H3K27me3 and the binding site of Kdm6b on the promoter of Laptm4b in mouse Lin$^-$c-Kit$^+$ cells
m-Laptm4b-5UTR-1-R	GAGAAGCCGTGGGTCAC	
h-LAPTM4B-prom-H3K27-1F	GGTGCACGCTGATGGATTTA	ChIP-qPCR primers to validate the modified sites of H3K27me3 and the binding site of KDM6B on the promoter of LAPTM4B in human cell lines
h-LAPTM4B-prom-H3K27-1R	GTGACCCGAGTCCGTGA	
h-LAPTM4B-prom-H3K27-2F	ATTTACTCACCGGGTGCTTG	
h-LAPTM4B-prom-H3K27-2R	GGTACCCTTCGCTCCGA	
shmLaptm4b-744-1F	aaaaaTGAATCCTACCTGTTTGGTCCTTATtttttAGAGTGGTCT	shRNA primers to knock down Laptm4b in mouse Lin$^-$c-Kit$^+$ cells using FAMSi system.
shmLaptm4b-744-2F	ggtGGTCTCGggcaaaaaaTGAATCCTACCTGTTTGGTCCTTAT	
shmLaptm4b-803-1R	aaaaaAAACGCAGCTAATCAAGTAGCCCTTtttttTTCGTCCTTTC	
shimLaptm4b-803-2R	ggtGGTCTCGgccaaaaaaAAACGCAGCTAATCAAGTAGCCCTT	

Supplemental Table 3: Overlapped genes in Figure 6B

Hebp2	Arfgef3	Fbxo27
Chrna1os	Laptm4b	Cmtm8
Prss16	Timp4	Mlc1
Smim10l2a	Dnah7a	Hif3a
Slc4a5	Myh7b	Cplx4
Inf2	Fam20c	Ckm
Fbp1	Glt8d2	Slamf1
Gcat	Them7	Cyp27a1
