# Supplementary material for: Evi1 governs Kdm6b-mediated histone demethylation to regulate the Laptm4b-driven mTOR pathway in hematopoietic progenitor cells
Source: J Clin Invest. 2024 Dec 16;134(24):e173403. doi: 10.1172/JCI173403 (PMC11645144; doi:10.1172/JCI173403)
Supplement: Unedited blot and gel images [file jci-134-173403-s138.pdf]

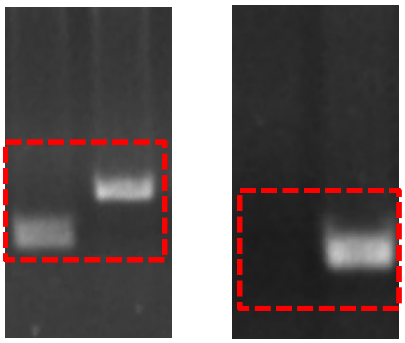

Full unedited gel image. Red boxes indicate the images used in Figure 1B.

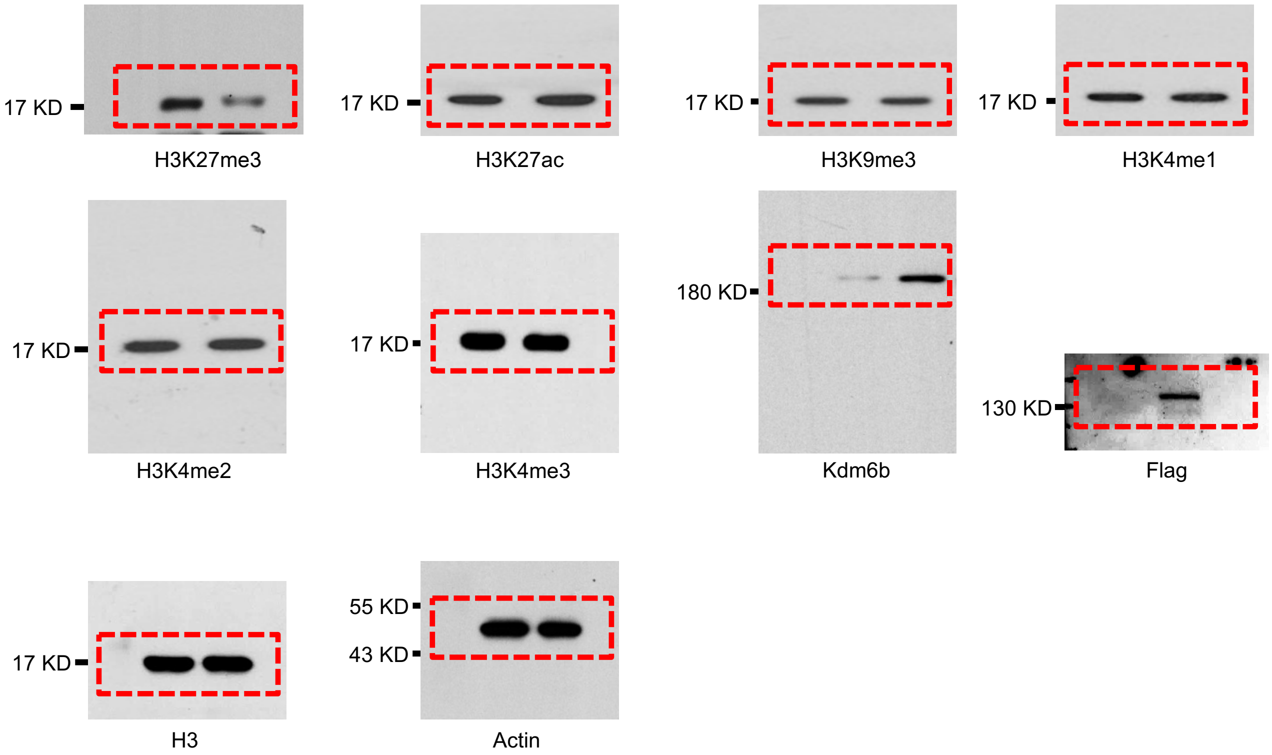

Full unedited Western blot. Red boxes indicate the images used in Figure 5A.

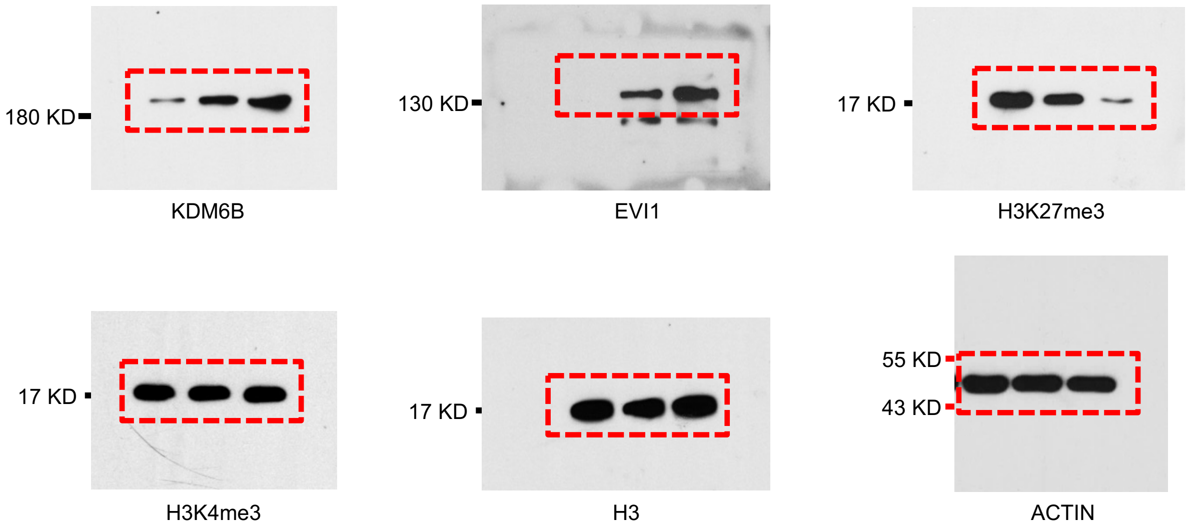

Full unedited Western blot. Red boxes indicate the images used in Figure 5G.

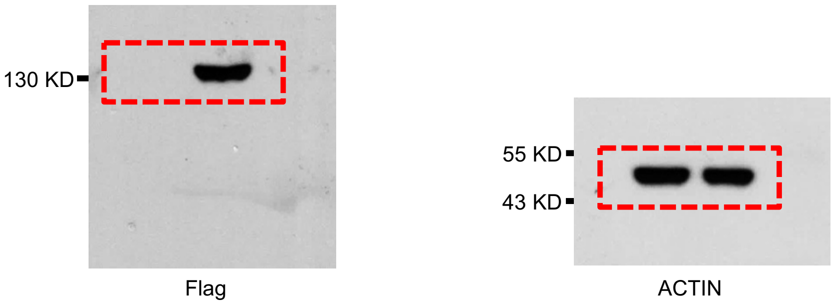

Full unedited Western blot. Red boxes indicate the images used in Figure 5H.

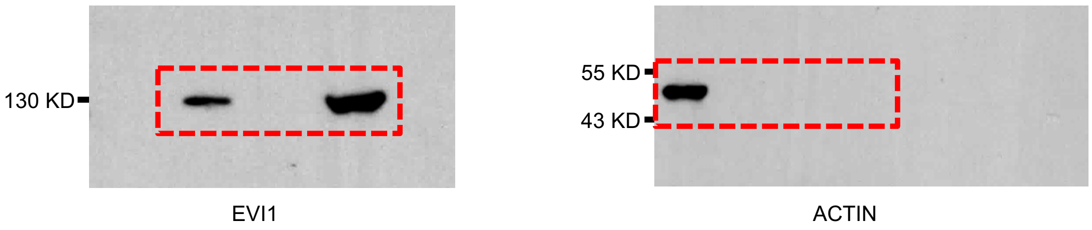

Full unedited Western blot. Red boxes indicate the images used in Figure 5J.

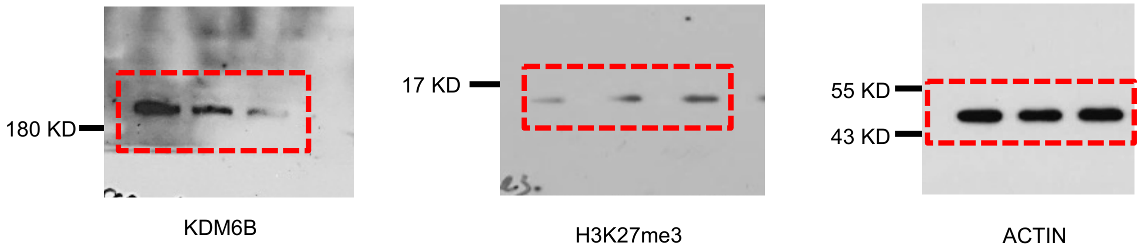

Full unedited Western blot. Red boxes indicate the images used in Figure 5J.

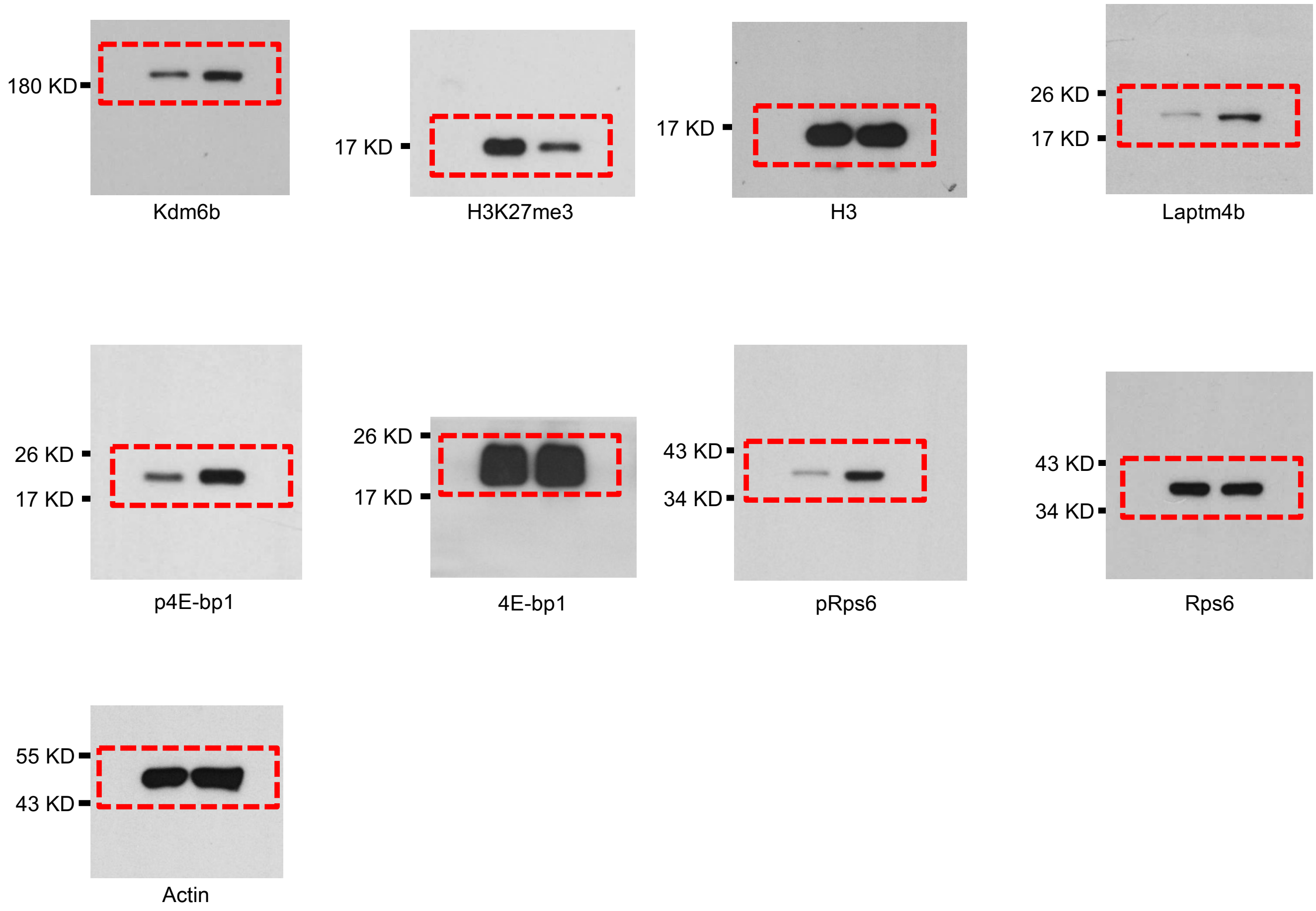

Full unedited Western blot. Red boxes indicate the images used in Figure 7A.

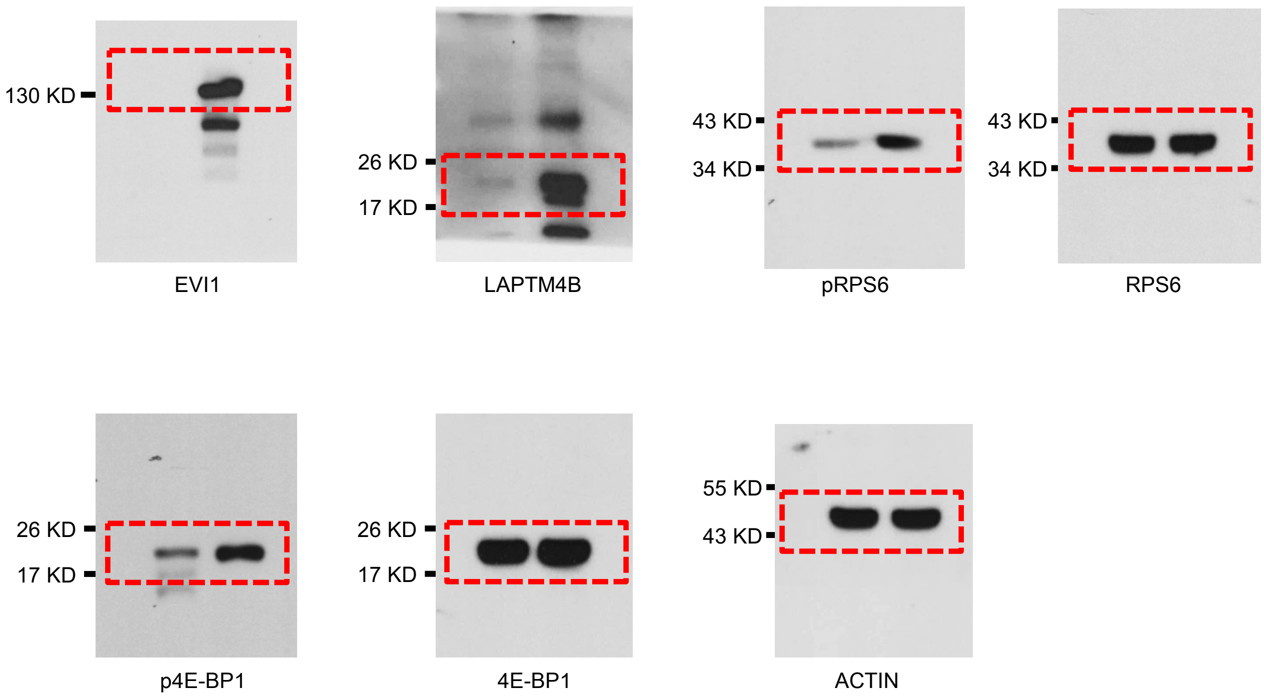

Full unedited Western blot. Red boxes indicate the images used in Figure 7B.

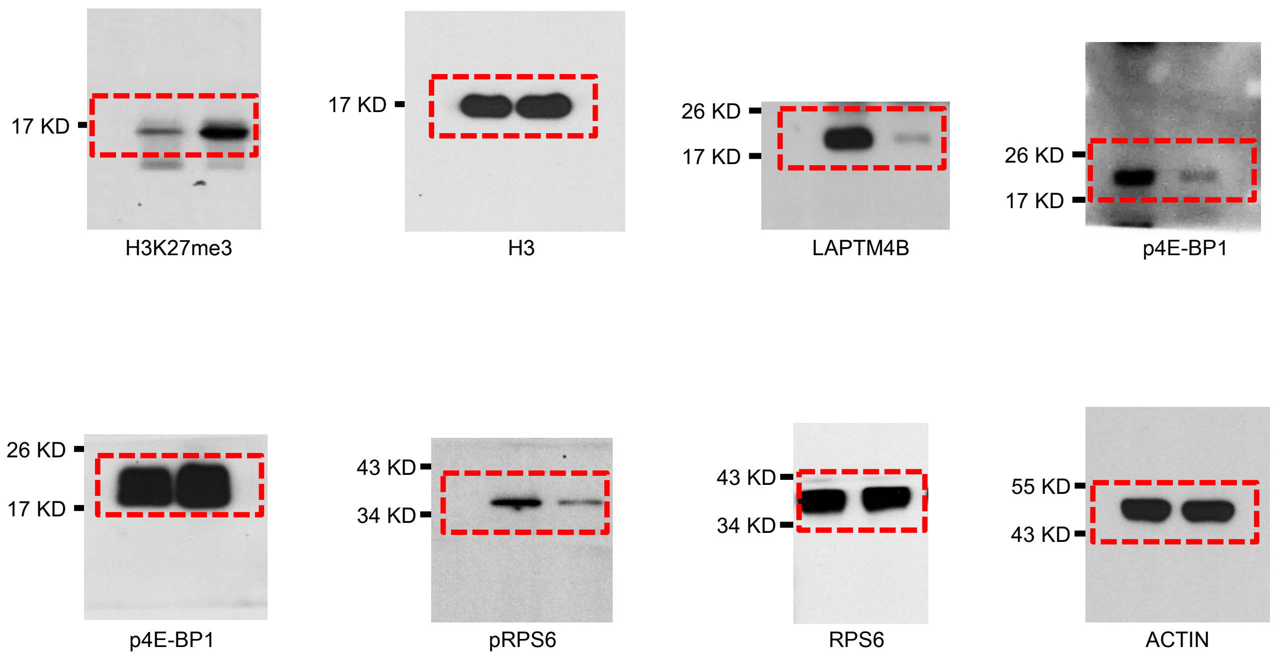

Full unedited Western blot. Red boxes indicate the images used in Figure 7C.

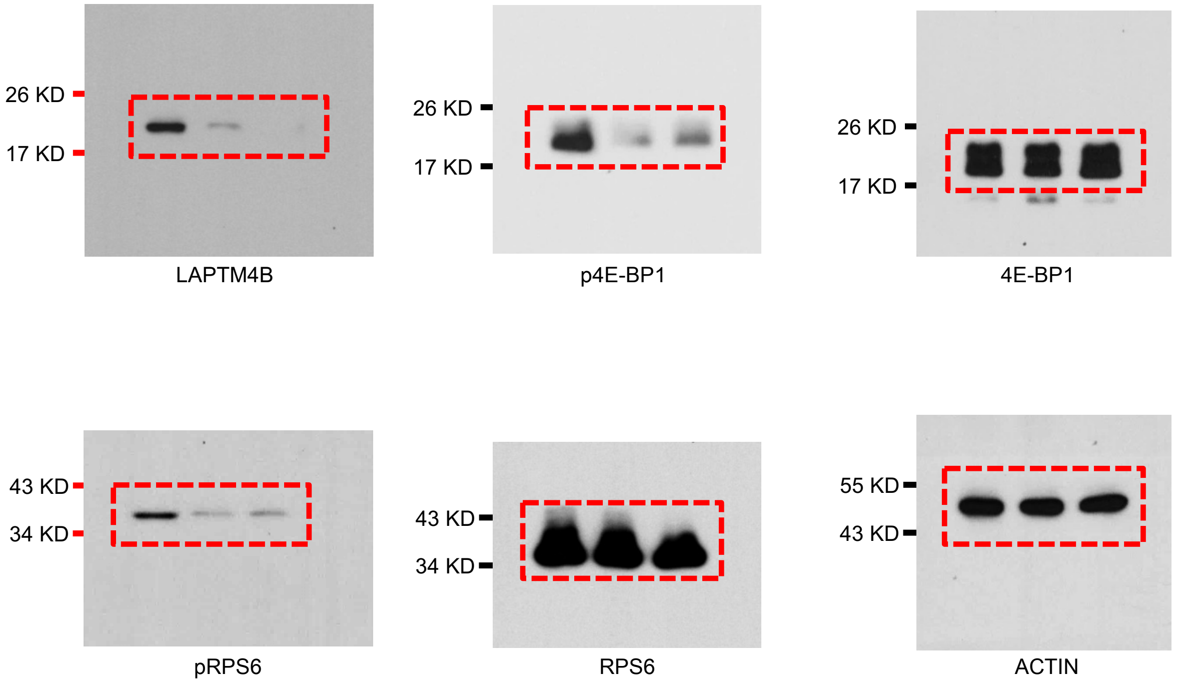

Full unedited Western blot. Red boxes indicate the images used in Figure 7D.

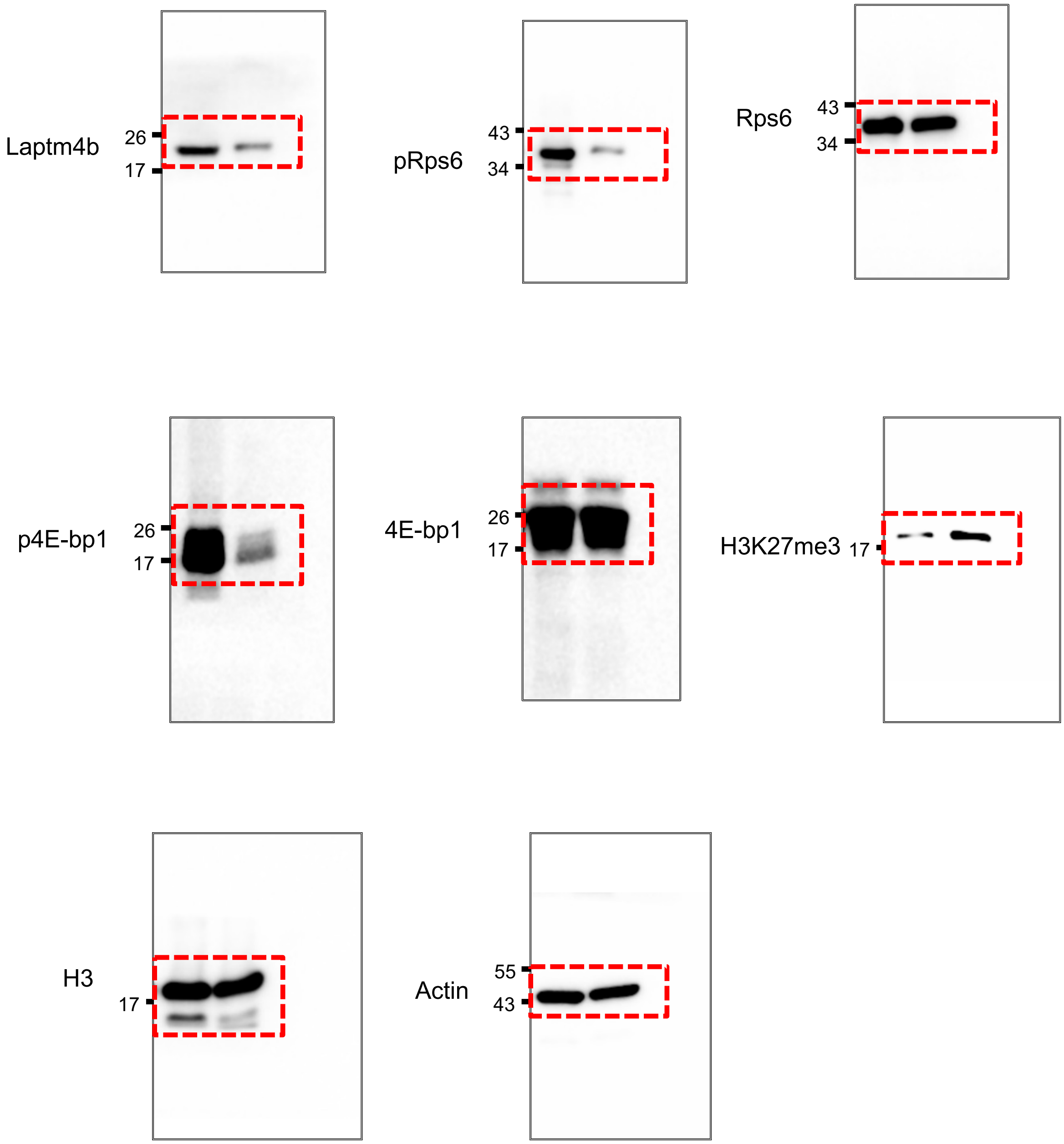

Full unedited Western blot. Red boxes indicate the images used in Figure 7E.
